# Posicionamento Luso-Brasileiro de Emergências Hipertensivas – 2020

**DOI:** 10.36660/abc.20190731

**Published:** 2020-05-12

**Authors:** José Fernando Vilela-Martin, Juan Carlos Yugar-Toledo, Manuel de Carvalho Rodrigues, Weimar Kunz Sebba Barroso, Luís Carlos Bronze S. Carvalho, Francisco José Torres González, Celso Amodeo, Vitor Manuel Margarido Paixão Dias, Fernando Carvalho Moreira Pinto, Luís Filipe Reis Martins, Marcus Vinícius Bolívar Malachias, Paulo Cesar Veiga Jardim, Dilma do Socorro Moraes de Souza, Oswaldo Passarelli, Eduardo Costa Duarte Barbosa, Jorge Junqueira Polonia, Rui Manoel dos Santos Póvoa

**Affiliations:** 1 Faculdade Estadual de Medicina de São José do Rio Preto São José do Rio Preto SP Brasil Faculdade Estadual de Medicina de São José do Rio Preto (FAMERP), São José do Rio Preto, SP – Brasil; 2 Centro Hospitalar Universitário Cova da Beira Covilhã Portugal Centro Hospitalar Universitário Cova da Beira, Covilhã – Portugal; 3 Universidade da Beira Interior Covilhã Portugal Universidade da Beira Interior, Covilhã – Portugal; 4 Liga de Hipertensão Arterial Universidade Federal de Goiás Goiânia GO Brasil Liga de Hipertensão Arterial da Universidade Federal de Goiás (UFG), Goiânia, GO – Brasil; 5 Marinha Portuguesa Lisboa Portugal Marinha Portuguesa, Lisboa – Portugal; 6 Vithas Hospital Nisa 9 de Octubre Valencia Espanha Vithas Hospital Nisa 9 de Octubre,Valencia – Espanha; 7 Universidade Federal de São Paulo São Paulo SP Brasil Universidade Federal de São Paulo (UNIFESP), São Paulo, SP – Brasil; 8 Centro Hospitalar de Vila Nova Gaia Espinho Portugal Centro Hospitalar de Vila Nova Gaia, Espinho – Portugal; 9 Centro Hospitalar de Entre o Douro e Vouga Santa Maria da Feira Portugal Centro Hospitalar de Entre o Douro e Vouga, E.P.E., Santa Maria da Feira – Portugal; 10 Hospital Escola Universidade Fernando Pessoa Cosme Portugal Hospital Escola da Universidade Fernando Pessoa, Cosme – Portugal; 11 Faculdade de Ciências Médicas de Minas Gerais Belo Horizonte MG Brasil Faculdade de Ciências Médicas de Minas Gerais, Belo Horizonte, MG – Brasil; 12 Universidade Federal de Goiás Goiânia GO Brasil Universidade Federal de Goiás (UFG), Goiânia, GO – Brasil; 13 Hospital do Coração De Goiás Goiânia GO Brasil Hospital do Coração De Goiás, Goiânia, GO – Brasil; 14 Faculdade de Medicina Universidade Federal do Pará Belém PA Brasil Faculdade de Medicina da Universidade Federal do Pará (UFPA), Belém, PA – Brasil; 15 Instituto Dante Pazzanese de Cardiologia São Paulo SP Brasil Instituto Dante Pazzanese de Cardiologia,São Paulo, SP – Brasil; 16 Liga de Combate à Hipertensão de Porto Alegre Porto Alegre RS Brasil Liga de Combate à Hipertensão de Porto Alegre, Porto Alegre, RS – Brasil; 17 Faculdade de Medicina Universidade do Porto Porto Portugal Faculdade de Medicina da Universidade do Porto, Porto – Portugal

**Table:** 

Declaração de potencial conflito de interesses dos autores/colaboradores do Posicionamento Luso-Brasileiro de Emergências Hipertensivas – 2020 Se nos últimos 3 anos o autor/colaborador do Posicionamento:							

Nomes Integrantes do Posicionamento	Participou de estudos clínicos e/ou experimentais subvencionados pela indústria farmacêutica ou de equipamentos relacionados à diretriz em questão	Foi palestrante em eventos ou atividades patrocinadas pela indústria relacionados à diretriz em questão	Foi (é) membro do conselho consultivo ou diretivo da indústria farmacêutica ou de equipamentos	Participou de comitês normativos de estudos científicos patrocinados pela indústria	Recebeu auxílio pessoal ou institucional da indústria	Elaborou textos científicos em periódicos patrocinados pela indústria	Tem ações da indústria
Celso Amodeo	Medtronic	Não	Não	Não	Novonordisk, Pfizer, Sankyo	Medley	Não
Dilma do Socorro Moraes de Souza	Não	Não	Não	Não	Não	Não	Não
Eduardo Costa Duarte Barbosa	Não	Servier, EMS	Não	Não	Servier, EMS, Torrent	EMS, Medley, Novartis	Não
Fernando Carvalho Moreira Pinto	Não	Não	Não	Não	Não	Não	Não
Francisco José Torres González	Não	Não	Não	Não	Não	Não	Não
Jorge Junqueira Polonia	Não	Não	Não	Não	Não	Não	Não
Jose Fernando Vilela-Martin	Não	Não	Não	Não	Não	Não	Não
Juan Carlos Yugar-Toledo	Não	Não	Não	Não	Não	Não	Não
Luís Carlos Bronze S. Carvalho	Não	Não	Não	Não	Não	Não	Não
Luís Filipe Reis Martins	Não	Não	Não	Não	Não	Não	Não
Manuel de Carvalho Rodrigues	Não	Não	Não	Não	Não	Não	Não
Marcus Vinícius Bolivar Malachias	Não	Libbs, Biolab	Não	Não	Não	Libbs, Biolab, Aché	Não
Oswaldo Passarelli Júnior	Não	Não	Não	Não	Não	Não	Não
Paulo César Veiga Jardim	Não	Não	Não	Não	Não	Biolab, Aché, Libbs	Não
Rui Manoel dos Santos Póvoa	Não	Não	Não	Não	Não	Não	Não
Vitor Manuel Margarido Paixão Dias	Não	Não	Não	Não	Servier, Tecnimede	Não	Não
Weimar Kunz Sebba Barroso	Boehringer, Torrent, EMS, Amgen, AstraZeneca, Novartis	EMS, Servier, Medley, Omron, Cardios	Omron	Não	EMS, Servier	EMS, Servier, Medley	Não

## Sumário

1. Definição, Epidemiologia e Classificação das Emergências Hipertensivas 738

2. Aspectos Fisiopatológicos da Emergência Hipertensiva 739

2.1. Autorregulação do Fluxo Sanguíneo Cerebral 739

3. Avaliação Clínica e Laboratorial 740

4. Tratamento das Emergências Hipertensivas: Princípios Gerais, Principais Fármacos e Dosagens 740

5. Encefalopatia Hipertensiva 741

5.1. Manifestações Clínicas 741

5.2. Diagnóstico 741

5.3. Tratamento 742

6. Hipertensão Maligna ou Acelerada 742

7. Acidente Vascular Cerebral e Emergência Hipertensiva 743

7.1. Acidente Vascular Cerebral Isquêmico 743

7.2. Acidente Vascular Cerebral Hemorrágico 744

8. Síndromes Coronarianas Agudas e Emergência Hipertensiva 744

9. Disfunção Ventricular Esquerda Aguda na Emergência Hipertensiva 745

10. Síndromes Aórticas Agudas 745

10.1. Tratamento 745

11. Emergências Hipertensivas na Gestação 745

11.1. Tratamento 746

12. Emergências Adrenérgicas 746

13. Drogas Ilícitas e Emergência Hipertensiva 747

14. Emergência Hipertensiva no Pós-Operatório de Cirurgia Vascular 748

Referências 748

## 1. Definição, Epidemiologia e Classificação das Emergências Hipertensivas

A emergência hipertensiva (EH) está integrada em um quadro nosológico mais geral denominado crise hipertensiva (CH). A CH representa situações clínicas que cursam com elevação aguda da pressão arterial (PA), geralmente níveis de PA sistólica (PAS) ≥ 180 mmHg e diastólica (PAD) ≥ 120 mmHg, que podem resultar ou não em **lesões de órgãos-alvo** (LOA) (coração, cérebro, rins e artérias).^1 - 5^ A CH pode se apresentar sob duas formas distintas em relação à gravidade e ao prognóstico: a urgência hipertensiva (UH) e a EH. Casos de EH cursam com elevação acentuada da PA associada a LOA e risco imediato de morte, fato que requer redução rápida e gradual dos níveis tensionais em minutos a horas, com monitoramento intensivo e uso de fármacos por via endovenosa (EV).^1 - 5^ Ela pode se manifestar como um evento cardiovascular, cerebrovascular, renal ou na gestação, na forma de pré-eclâmpsia ou eclâmpsia. Embora a definição clássica das duas apresentações da CH a descreva com valores acima dos 180/120 mmHg, atualmente o maior consenso se estabelece no conceito de que mais do que os valores é o dano ou o risco iminente de acometimento de órgãos-alvo que distingue a EH da UH. Assim, a UH caracteriza-se por elevações da PA, sem LOA e sem risco de morte iminente, fato que permite redução mais lenta dos níveis de PA em período de 24 a 48 horas. Atualmente, existe uma ampla discussão sobre a real existência do diagnóstico “urgência hipertensiva”.^6^ Muitos preconizam que esta classificação necessita ser atualizada (se não abandonada) e que a maior importância diagnóstica é a observação dos sinais/sintomas e da disfunção aguda dos órgãos-alvo, mais do que no valor da PA. Outros acreditam que o termo correto deveria ser “elevação da PA sem LOA em evolução”.^5 , 7^

Como visto, embora o nível de PA seja frequentemente muito elevado (≥ 180/120 mmHg), não é isso que define EH, mas o comprometimento dos órgãos-alvo. Portanto, o padrão numérico que define a CH é conceitual e serve como parâmetro de conduta, mas não deve ser usado como critério absoluto.

Se a definição de CH hoje está mais universalmente aceita, a epidemiologia e prevalência desta condição são ainda questões de baixo conhecimento da comunidade científica. Na literatura, existem poucos estudos sobre o tema e todos eles com número limitado de participantes. Atualmente, discute-se a hipótese de a não adesão ao tratamento ser um dos fatores mais prevalentes na etiologia da CH, sem especificações quanto à separação entre UH e EH. Nos EUA, nos maiores estudos seriados a incidência de CH é de cerca de 4,8%, sendo 0,8% atribuída às EH.^8 , 9^ Outros centros mostram que a CH responde por uma taxa variável de 0,45 a 0,59% de todos os atendimentos de emergência hospitalar e 1,7% das emergências clínicas, sendo a UH mais comum do que a EH.^10 - 12^ Acidente vascular cerebral (AVC) isquêmico e edema agudo de pulmão (EAP) são as situações clínicas mais encontradas nas EH.^10 , 11^ Estima-se que cerca de 1% dos indivíduos hipertensos possa vir a apresentar uma CH ao longo da sua vida.^1 , 2^ As situações clínicas envolvidas em uma EH, de acordo com as LOA, são mostradas na Tabela 1 . A Tabela 2 mostra as principais situações relacionadas à UH.

**Tabela 1:** – Situações que cursam com lesões em órgãos-alvo caracterizando emergências hipertensivas1-5

Hipertensão grave associada a complicações agudas
**Eventos cerebrovasculares**
- Encefalopatia hipertensiva
- Hemorragia intracerebral
- Hemorragia subaracnoide
- AVC isquêmico
**Eventos cardiocirculatórios**
- Dissecção aguda de aorta
- Edema agudo de pulmão com insuficiência ventricular esquerda
- Infarto agudo do miocárdio
- Angina instável
**Doença renal**
- Insuficiência renal rapidamente progressiva

**Crises adrenérgicas graves**

- Crise do feocromocitoma
- Superdosagem de drogas ilícitas (cocaína, *crack* , LSD)

**Hipertensão na gestação**

- Eclâmpsia
- Pré-eclâmpsia grave
- Síndrome “HELLP”
- Hipertensão grave em final de gestação

*HELLP: hemólise, enzimas hepáticas elevadas e plaquetopenia; AVC: acidente vascular cerebral; LSD: dietilamida do ácido lisérgico.*

**Tabela 2:** – Situações que cursam com urgências hipertensivas1-5

Hipertensão grave associada a:
- Insuficiência coronariana
- Insuficiência cardíaca
- Aneurisma de aorta
- Acidente vascular cerebral não complicado
- Epistaxe grave
- Queimaduras extensas
- Estados de hipocoagulabilidade

**Vasculites sistêmicas**

- Peri-operatório
- Pré-operatório em cirurgias de urgência
- Intraoperatório (cirurgias cardíacas, vasculares, neurocirurgias, feocromocitoma etc.)
- Hipertensão estágio III no pós-operatório (transplante de órgão, cirurgias cardíacas, vasculares, neurocirurgias etc.)

**Crises adrenérgicas leves/moderadas**

- Síndrome do rebote (suspensão súbita de inibidores adrenérgicos)
- Interação medicamentoso-alimentar (tiramina vs. inibidores da MAO)
- Consumo excessivo de estimulantes (anfetaminas, tricíclicos etc.)

**Na gestação**

- Pré-eclâmpsia
- Hipertensão estágio III

*MAO: monoaminoxidase.*

## 2. Aspectos Fisiopatológicos da Emergência Hipertensiva

A fisiopatogenia da EH não é completamente elucidada. De forma geral, dois mecanismos diferentes podem desempenhar papéis centrais em sua fisiopatogênese. O primeiro é o desequilíbrio no sistema de autorregulação do leito vascular, que cursa com redução da pressão de perfusão, com consequente diminuição do fluxo sanguíneo e aumento da resistência vascular, originando estresse mecânico e lesão endotelial.^13^ O segundo mecanismo é a ativação do sistema renina-angiotensina, levando a uma maior vasoconstrição, produzindo um ciclo vicioso de lesão endotelial, necrose fibrinoide de arteríolas e subsequente isquemia.^14^ A lesão vascular provoca deposição de plaquetas e fibrina, caracterizando também o estado protrombótico.^15^ Posterior isquemia resultante estimula a liberação de mais substâncias vasoativas, criando um círculo vicioso.

### 2.1. Autorregulação do Fluxo Sanguíneo Cerebral

O conhecimento do mecanismo de autorregulação do fluxo sanguíneo para os órgãos-alvo (fluxo cerebral, coronariano e renal) é vital para uma melhor conduta anti-hipertensiva nos casos de EH. A autorregulação do fluxo sanguíneo cerebral (FSC) é mantida pela relação entre pressão de perfusão cerebral (PPC) e resistência cerebrovascular (RCV), isto é, FSC = PPC/RCV (PPC = pressão arterial média − pressão venosa média). PPC é a diferença entre a PA, que ajuda na irrigação dos tecidos, e a pressão venosa. Sob PPC normal, a pressão venosa não é importante, de modo que a PPC é equivalente à PA. Reduções na PPC podem ser causadas por reduções na PA ou aumento da pressão intracraniana (PIC), o que aumenta a pressão venosa. Elevações na PIC podem ocorrer como resultado de doença oclusiva arterial ou venosa ou hemorragia intracerebral. Em indivíduos normotensos, uma ampla variação na PA (entre 60 e 150 mmHg) pode ocorrer sem alterar o FSC. Um aumento na PPC (ou PA) provoca elevação na RCV, protegendo assim o paciente contra o edema cerebral, e reduções na PPC causam queda na RCV, protegendo assim o paciente da isquemia tecidual. Quando a PPC exceder o limite superior de autorregulação, o FSC aumentará causando edema cerebral. Por outro lado, quando a PPC decair abaixo do limite inferior de autorregulação, o FSC diminuirá causando isquemia cerebral.^16 , 17^

Em indivíduos hipertensos, essa relação é alterada de tal forma que o limite inferior de autorregulação é maior do que em indivíduos normotensos. Portanto, diminuições inadequadas na PPC podem dificultar a irrigação tecidual e, consequentemente, agravar a área isquêmica viável. Por essa razão, é aconselhável reduzir, inicialmente, a pressão média em 20 a 25% em relação aos valores iniciais, pois estará próxima do limite inferior de autorregulação.^18^ Deve-se estar atento a essa situação, pois a maioria dos pacientes com EH é portadora de hipertensão crônica com desvio da curva de autorregulação de pressão/fluxo (cerebral, coronariano e renal) para a direita e não apresenta lesão aguda de órgão-alvo, motivo pelo qual uma redução súbita da PA pode estar associada à morbidade significativa.^18 - 20^

## 3. Avaliação Clínica e Laboratorial

Durante a abordagem da EH, o profissional deverá realizar a diferenciação entre emergência e urgência, fazendo o diagnóstico correto das diversas situações de EH, a fim de selecionar a terapia mais adequada para cada LOA. Isso é muito importante, pois o diagnóstico e o tratamento corretos podem evitar agravamento do quadro clínico decorrente da situação crítica. A abordagem aos pacientes com EH requer avaliação clínica e testes complementares realizados em centros de emergência clínica com suporte hospitalar. A PA deve ser aferida nos dois braços (no mínimo 03 medidas), preferencialmente em ambiente tranquilo. Indivíduos com elevações agudas da PA apresentam mais frequentemente alterações metabólicas, caracterizadas por hiperglicemia, dislipidemia, menores níveis de potássio e função renal reduzida.^21^ A sequência de etapas utilizadas no manejo dos pacientes com CH é a seguinte:^1 - 5 , 22 , 23^

1. Procurar fatores que possam ter desencadeado a elevação aguda da PA.
2. Investigar sintomas ou situações que simulam CH (cefaleia, labirintite, trauma físico, dor, estresse emocional, problemas familiares ou profissionais).
3. Observar história de hipertensão arterial sistêmica (HAS), tempo de evolução, uso de fármacos anti-hipertensivos (doses e adesão farmacológica).
4. Investigar episódios anteriormente ocorridos semelhantes à situação atual.
5. Investigar uso de medicamentos que possam interferir no controle da PA (anti-inflamatórios, esteroides, analgésicos, antidepressivos, moderadores de apetite).
6. Avaliar uso ou consumo abusivo de álcool e substâncias tóxicas (cocaína, *crack* , dietilamida do ácido lisérgico [LSD]).
7. Avaliar uso de inibidores adrenérgicos que foram subitamente interrompidos (clonidina, metildopa e betabloqueadores).
8. Observar se existe associação com outras morbidades e fatores de risco (diabetes, doença cardíaca, doença renal, tabagismo, dislipidemia).
9. A história clínica e o exame físico devem ser realizados de acordo com a presença de LOA:

- Sistema nervoso central (observar ocorrência de cefaleia, tontura, distúrbios visuais e da fala, nível de consciência, agitação ou apatia, confusão, déficits neurológicos focais, rigidez de nuca, convulsões e coma).
- Sistema cardiovascular (avaliar ritmo cardíaco, queixa de palpitações e sopro carotídeo, investigar dor e desconforto torácico e precordial, no abdome ou no dorso, além de sinais e sintomas de insuficiência ventricular esquerda – ritmo de galope, dispneia, estase venosa jugular, pulsos periféricos, saturação de oxigênio).
- Sistema renal e geniturinário (avaliar alterações no volume e na frequência miccional ou no aspecto da urina, desidratação, edema em membros inferiores, hematúria e disúria). Nota: não se esquecer de examinar o abdome (massas pulsáteis abdominais e sopro abdominal).
- Fundo de olho (observar se existe vasoespasmo, cruzamentos arteriovenosos, espessamento na parede arterial e aspecto em fio de cobre ou prata, exsudatos duros e moles, hemorragias e papiledema).

Os exames complementares deverão ser realizados conforme o envolvimento dos órgãos-alvo:

- Sistema nervoso central (tomografia computadorizada, ressonância magnética e punção lombar).
- Sistema cardiovascular (eletrocardiografia, radiografia de tórax, ecocardiografia, marcadores de necrose miocárdica, angiotomografia, ressonância magnética).
- Sistema renal (urina de rotina, ureia, creatinina, eletrólitos e gasometria).

## 4. Tratamento das Emergências Hipertensivas: Princípios Gerais, Principais Fármacos e Dosagens

As melhores condições diagnósticas e terapêuticas têm proporcionado grande redução na taxa de mortalidade em 1 ano, que variava de 80%, em 1928, a 50%, em 1955 e, a somente, 10% em 1989.^24 , 25^ O tratamento dos pacientes com quadro clínico de EH tem como propósito a redução rápida da PA com a finalidade de impedir a progressão das LOA. Portanto, eles devem ser admitidos em terapia intensiva, submetidos a tratamento anti-hipertensivo por via EV e monitorados cuidadosamente durante a terapia parenteral para evitar a ocorrência de hipotensão. As recomendações gerais de redução da PA sugeridas pelo *VII Joint National Committee* (JNC)^26^ para EH são sumarizadas da seguinte forma:

- ↓ PA ≤ 25% na primeira hora.
- ↓ PA 160/100 a 110 mmHg em 2 a 6h.
- PA 135/85 mmHg 24 a 48h.

Entretanto, EH devem ser abordadas considerando o sistema ou o órgão-alvo acometido. Assim, cada comprometimento orgânico da EH (cardiovascular, cerebral, renal e outros) deve ser caracterizado previamente antes de iniciar a terapia anti-hipertensiva específica (ver “Avaliação clínica e laboratorial”).

Atualmente, várias opções terapêuticas medicamentosas estão disponíveis para o tratamento das EH. O fármaco anti-hipertensivo ideal para uso parenteral deve apresentar as seguintes características: capacidade de reverter alterações fisiopatológicas envolvidas, rápido início de ação, curva dose-resposta previsível, mínimo ajuste de dosagem, alta seletividade, não promover elevação da PIC, pronta reversibilidade, baixo risco de promover hipotensão arterial, fácil substituição por fármacos para uso oral e satisfatória relação custo-benefício. Na Tabela 3 são apresentadas sucintamente as propriedades farmacocinéticas e farmacodinâmicas dos principais fármacos anti-hipertensivos utilizados nas EH.^2 , 22 , 26 - 28^ No Brasil, os seguintes fármacos estão disponíveis para uso nas EH: nitroprussiato de sódio, nitroglicerina, labetalol, esmolol, metoprolol, hidralazina e enalaprilato.

**Tabela 3:** – Propriedades farmacocinéticas e farmacodinâmicas dos principais medicamentos anti-hipertensivos para uso por via parenteral

Fármacos	Modo de administração e dosagem	Início	Duração	Vantagens	Desvantagens
Nitroglicerina (vasodilatador arterial e venoso do doador de óxido nítrico)	Infusão contínua 5 a 15 mg/h	2 a 5 min	3 a 5 min	Perfusão coronariana	Cefaleia, eficácia variável, taquifilaxia
Nitroprussiato de sódio (vasodilatador arterial e venoso)	Infusão contínua 0,5 a 10 μg/kg/min	Imediato	1 a 2 min	Titulação	Intoxicação por tiocianato, hipotensão, náuseas, vômitos, espasmo muscular
Metoprolol (betabloqueador)	Ataque: 5 mg EV (repetir a cada 10 min, até 20 mg se necessário)	5 a 10 min	3 a 4 h	Redução do consumo de O_2_	Bradicardia, BAV, broncospasmo
Labetalol (alfa e betabloqueador)	Ataque: 20 a 80 mg a cada 10 min Infusão contínua 2 mg/min (máximo 300 mg/24 h)	5 a 10 min	2 a 6 h	Betabloqueador e vasodilatador	Náuseas, vômitos, BAV, broncospasmo, hipotensão ortostática
Esmolol (Betabloqueador ultrasseletivo de ação ultrarrápida)	Ataque: 500 μg/kg Infusão intermitente: 25 a 50 μg/kg/min ↑ 25 μg/kg/min a cada 10 a 20 min Máximo: 300 μg/kg/min	1 a 2 min	1 a 20 min	Betabloqueador seletivo	Bradicardia, BAV, broncospasmo
Hidralazina (vasodilatador de ação direta)	10 a 20 mg EV ou 10 a 40 mg IM a cada 6 h	10 a 20 min EV ou 20 a 30 min IM	3 a 12 h	Eclâmpsia ou eclâmpsia iminente	Taquicardia, cefaleia, vômitos. Piora da angina e do IAM. Cuidado com pressão intracraniana elevada
Enalaprilato (IECA)	Infusão intermitente: 1,25 a 5 mg a cada 6 h	15 min	4 a 6 h	ICC, IVE aguda	Hipotensão, insuficiência renal
Furosemida (diurético de alça)	Infusão	5 a 10 min	30 a 90 min	ICC, IVE	Hipopotassemia

*IAM: infarto agudo do miocárdio; ICC: insuficiência cardíaca congestiva; IVE: insuficiência ventricular esquerda; BAV: bloqueio atrioventricular; IECA: inibidor da enzima conversora da angiotensina; EV: via endovenosa; IM: via intramuscular.*

## 5. Encefalopatia Hipertensiva

Encefalopatia hipertensiva é uma disfunção neurológica definida por sinais e/ou sintomas de edema cerebral secundário à elevação súbita e/ou mantida da PA. Acontece em indivíduos hipertensos crônicos que desenvolvem HAS maligna ou naqueles previamente normotensos que podem apresentar elevações agudas da PA por outros mecanismos, cursando com falência dos mecanismos de autorregulação da perfusão cerebral. Representa um diagnóstico de exclusão, confirmado retrospectivamente quando o quadro neurológico melhora após o controle da PA.

### 5.1. Manifestações Clínicas

A encefalopatia hipertensiva pode se apresentar com início insidioso, evoluindo com cefaleia holocraniana, náuseas ou vômitos. Posteriormente, podem surgir alterações do estado mental e campimétricas, fotopsia, visão turva, alucinações visuais, crises convulsivas generalizadas, hiperreflexia e sinais de hipertensão intracraniana.^29 , 30^ No momento em que aparecem as manifestações neurológicas, geralmente, a PAD encontra-se acima de 125 mmHg. A resolução do quadro, quer do ponto de vista clínico, quer do ponto de vista de imagem, demora, em média, várias semanas após o controle da PA. A persistência de um déficit é sinal de existência de lesão neurológica focal.

### 5.2. Diagnóstico

A ressonância magnética é o exame diagnóstico de maior valor. Na sequência T2, evidenciam-se lesões hiperintensas na substância branca com comprometimento preferencial das regiões parieto-occipitais. O território irrigado pelo sistema vertebrobasilar pode ser atingido em casos mais graves. A presença de hipersinal ao coeficiente de difusão aparente sugere a ocorrência de edema vasogênico.^31^ Laboratorialmente, podem-se encontrar trombocitopenia, anemia hemolítica microangiopática, proteinúria, aumento dos valores da creatinina plasmática e das enzimas hepáticas. Na tomografia computadorizada, são habituais hipodensidades focais ou difusas na substância branca e no córtex, com sinais de edema. O eletroencefalograma mostra lentificação difusa com perda do ritmo alfa, ou atividade epileptiforme, se existirem crises convulsivas.

### 5.3. Tratamento

O objetivo é reduzir a PA média em aproximadamente 10 a 15% na primeira hora e não mais do que 25% ao fim do primeiro dia de tratamento. Diminuições mais profundas e rápidas podem provocar hipoperfusão cerebral e perda dos mecanismos de autorregulação vascular.^32 , 33^ Devido à necessidade de controle rápido da PA, recomenda-se o uso de fármacos por via EV, utilizando-se mais frequentemente nitroprussiato de sódio (vasodilatador arterial e venoso), nicardipina (bloqueador dos canais de cálcio di-hidropiridínico com ação vasodilatadora arteriolar), clevidipina (bloqueador dos canais de cálcio di-hidropiridínico de curta ação), labetalol (bloqueador alfa e beta-adrenérgico) ou fenoldopam (agonista dos receptores periféricos de dopamina-1). Na gestação, recomendam-se sulfato de magnésio, diazóxido ou hidralazina. Podem ser usados também corticoides (dexametasona), manitol (pode ser usado, se não houver doença renal) e anticonvulsivantes (em caso de crises).^23 , 30^ Nas primeiras 24 a 48 horas, devem-se introduzir fármacos de ação oral para melhor controle da PA (bloqueadores do sistema renina-angiotensina-aldosterona e bloqueadores dos canais de cálcio), com redução gradual da PAD para valores inferiores a 90 mmHg nos dois a três meses seguintes.^1 , 2 , 5 , 22^

## 6. Hipertensão Maligna ou Acelerada

**Figura 1:**
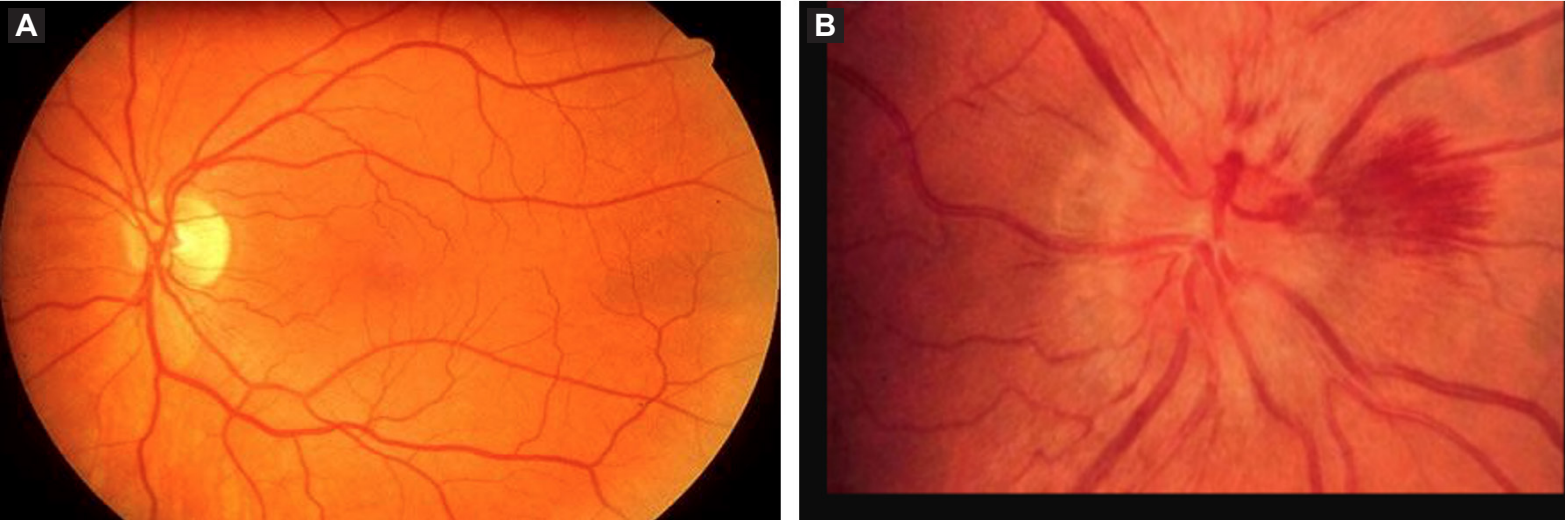
– *Fundo de olho normal (A). Fundo de olho de indivíduo com hipertensão maligna e papiledema (B).*

**Figura 2:**
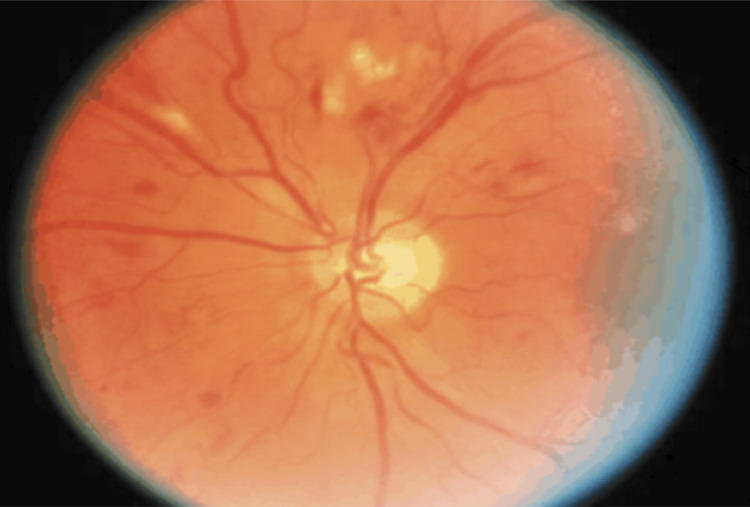
– *Exame de fundo de olho mostra papilas normais com estreitamento arteriolar difuso, focos de hemorragias superficiais e microaneurismas (retinopatia hipertensiva grau III da classificação de Keith-Wagener-Barker).*

**Figura 3:**
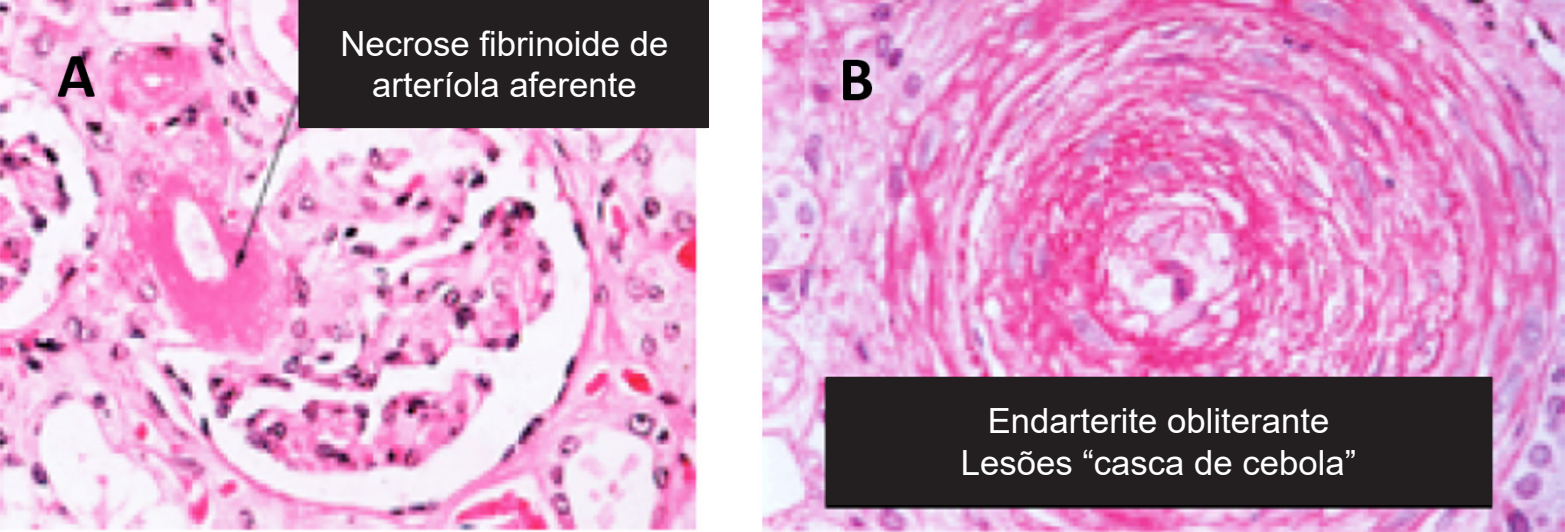
– *Lesões anatomopatológicas típicas de hipertensão arterial acelerada-maligna. Necrose fibrinoide de arteríola aferente (seta) (A). Endarterite obliterante (lesões em “casca de cebola”) (B).*

A hipertensão arterial maligna é caracterizada por apresentar HAS em níveis variados, mas em geral se apresenta com PA muito elevada (estágio 3), retinopatia com papiledema e LOA (rins e coração) rapidamente progressiva, e comumente cursa com evolução fatal, se não houver intervenção terapêutica ( Figura 1 ). A grave elevação da PA na presença de hemorragias retinianas e exsudatos ao fundo de olho, mas sem papiledema, é denominada hipertensão arterial acelerada ( Figura 2 ). Após a demonstração de que os achados clínicos e o prognóstico dessas duas formas de hipertensão eram semelhantes,^34^ os termos “maligna” e “acelerada” foram considerados intercambiáveis, de tal forma que a Organização Mundial da Saúde usa atualmente o termo acelerada-maligna para definir essa complicação. Caracteristicamente, a hipertensão maligna apresenta alterações vasculares sistêmicas que afetam principalmente os rins (a chamada nefrosclerose maligna), envolvendo basicamente dois processos: (a) endarterite proliferativa em pequenas e grandes arteríolas, com espessamento intimal, fragmentação e reduplicação da lâmina elástica interna e proliferação do músculo liso; a progressão dessa lesão, cuja aparência lembra “casca de cebola”, pode acarretar oclusão do lúmen do vaso e consequente redução do fluxo sanguíneo renal; (b) alteração necrosante das arteríolas, principalmente no hilo glomerular, sendo a parede dos vasos refeita com material granular eosinofílico que exibe as características de fibrina (necrose fibrinoide), causando destruição da morfologia normal e profundo estreitamento do lúmen. Essas alterações podem ocorrer em outros órgãos além dos rins e são as principais responsáveis pelas complicações fatais da doença ( Figura 3 ).^35^ O prognóstico da hipertensão maligna é quase sempre fatal, se não reconhecida ou não devidamente tratada precocemente e, no passado, a mortalidade chegava a 80% em dois anos.^36^ No entanto, desde a introdução do tratamento anti-hipertensivo, estudos têm mostrado que a sobrevida do indivíduo com hipertensão maligna melhorou muito.^37 - 39^ Em publicação com quase 500 pacientes de Birmingham (Reino Unido), os autores relataram uma melhora significativa da sobrevida de 5 anos, de 32% antes de 1977 para 91% em pacientes diagnosticados entre 1997 e 2006.^38^ O controle do paciente com hipertensão maligna habitualmente inclui o uso de 4 classes de fármacos, e as complicações hipertensivas podem se estabilizar e, em alguns casos, até ser revertidas.

## 7. Acidente Vascular Cerebral e Emergência Hipertensiva

O AVC pode se apresentar como uma EH. No indivíduo hipertenso crônico verifica-se um desvio para a direita da curva de autorregulação do FSC, fato que leva os hipertensos crônicos a tolerarem valores de PA substancialmente mais altos sem desenvolver encefalopatia. Quando os valores tensionais em hipertensos crônicos são reduzidos de forma agressiva e rápida, estes podem apresentar sintomas de hipoperfusão cerebral, mesmo se os valores de PA estiverem na faixa de autorregulação, como observado em indivíduos normotensos. Finalmente, os doentes com hipertensão grave podem perder a capacidade de autorregulação, aumentando o risco de isquemia cerebral quando a PA é reduzida de forma intempestiva.^16 - 18^

### 7.1. Acidente Vascular Cerebral Isquêmico

No AVC isquêmico, são recomendadas reduções cuidadosas da PA na ordem de 10 a 15% ao fim da primeira hora após instituição da terapêutica e apenas se PAS > 220 mmHg ou PAD > 120 mmHg.^40^ Se PAS estiver entre 180 a 230 mmHg ou PAD entre 105 a 120 mmHg e o paciente não for submetido à trombólise, recomenda-se a seguinte terapêutica: labetalol 10 mg EV, seguido de infusão contínua na dose de 2 a 8 mg/min; ou nicardipina nas doses referidas até se obter o efeito desejado. Se a PA persistir descontrolada ou PAD > 140 mmHg, deve-se considerar o uso do nitroprussiato de sódio por via EV.^40^

Nos casos de indivíduos com PA elevada e indicação para terapêutica trombolítica com alteplase, a PA deve ser cuidadosamente reduzida de tal forma que a PAS seja < 185 mmHg e a PAD < 110 mmHg antes do trombolítico ser administrado. Se a PA permanecer acima de 185/110 mmHg, a terapêutica trombolítica não deverá ser administrada.^40^ O labetalol é a primeira escolha, sendo a nicardipina a terapêutica alternativa. Recomenda-se a dose de labetalol de 10 a 20 mg por via EV durante 1 a 2 minutos (pode ser repetida por uma vez). A dose de nicardipina recomendada é 5 mg/h por via EV, com titulação da dose de 2,5 mg/h a cada 5 a 5 minutos (dose máxima 15 mg/h). Durante ou após trombólise ou outra terapia de reperfusão, a PA deve ser mantida em valores iguais ou inferiores a 180/105 mmHg.^40^

### 7.2. Acidente Vascular Cerebral Hemorrágico

No AVC hemorrágico, as metas para o tratamento constituem motivo de controvérsia.^41 - 43^ Durante a hemorragia intracerebral aguda, a elevação da PA é comum e está associado a maior risco de expansão do hematoma, aumento do risco de morte e pior prognóstico para a recuperação. Nesse caso, a diminuição imediata da PA (dentro de 6 horas) para valores <140/90 mmHg não mostrou benefício no desfecho primário de incapacidade ou morte aos 3 meses, apesar de reduzir a expansão do hematoma e melhorar a recuperação funcional.^41^ Por outro lado, outro estudo mostrou que a redução mais intensiva da PAS não apresentou benefício e se associou a maior número de eventos adversos renais.^42^ Assim, em indivíduos com AVC hemorrágico, as diretrizes europeias afirmam que a redução imediata da PA não é aconselhada para pacientes com PAS < 220 mmHg.^44^ Em indivíduos com PAS ≥ 220 mmHg, redução cuidadosa da PA com terapia EV, com objetivo de se atingir PAS < 180 mmHg, deve ser considerada.^44^ O labetalol, nas doses referidas, é a primeira escolha, sendo o nitroprussiato de sódio e a nicardipina as terapêuticas alternativas.^1 - 4 , 28^

## 8. Síndromes Coronarianas Agudas e Emergência Hipertensiva

Dados epidemiológicos indicam que a síndrome coronariana aguda (SCA) é a principal causa de morte e hospitalização em pacientes com EH. Além disso, quase 50% de todos os pacientes hipertensos admitidos na sala de emergência morreram de infarto agudo do miocárdio (IAM) durante o acompanhamento a longo prazo. Notavelmente, não foram encontradas diferenças para presença de outros fatores de risco, como tabagismo ou *diabetes mellitus* .^11 , 45^ Obviamente, a HAS associa-se a eventos coronarianos agudos como fator de risco, fator aterogênico e fator hemodinâmico com profundos efeitos em morbidade e mortalidade cardiovascular. Durante uma EH, o aumento da PA provoca estresse mecânico e lesão endotelial, causando aumento da permeabilidade vascular, ativação da cascata da coagulação, plaquetas, deposição de fibrina e trombose. Esse processo resulta em isquemia e liberação de mediadores vasoativos, criando um ciclo vicioso de lesão permanente. A ativação do sistema renina-angiotensina leva à maior vasoconstrição e à produção de citocinas pró-inflamatórias (fator de necrose tumoral [TNF]-alfa, interleucina [IL]-6 etc.). Além disso, aumenta a atividade da NADPH ( *nicotinamide adenine dinucleotide phosphate oxidase* ) e a produção de espécies reativas de oxigênio, causando estresse oxidativo. Esses mecanismos promovem hipoperfusão, isquemia miocárdica e disfunção endotelial, que se manifestam durante a EH.^14 , 15^

A avaliação do risco cardiovascular e a investigação das comorbidades são essenciais na abordagem do paciente em EH com quadro de SCA. O eletrocardiograma é o padrão-ouro para detecção de isquemia ou evento coronariano agudo. Além disso, sinais vitais (PA, saturação de oxigênio e frequência cardíaca) devem ser medidos com cuidado durante o exame físico de um paciente com EH. A análise laboratorial inclui a quantificação de enzimas cardíacas e determinação de troponina-I. Em um estudo retrospectivo, pacientes com CH e concentração elevada de c-troponina-I (cTn-I) apresentaram risco 2,7 vezes maior de eventos cardiovasculares adversos e AVC em dois anos de acompanhamento comparados àqueles com valores normais de cTn-I.^46^

O tratamento da EH associada à SCA deve ser iniciado com infusão de nitroglicerina. A nitroglicerina é um venodilatador que reduz a pré-carga e diminui a demanda cardíaca de oxigênio. Este agente é usado sobretudo em SCA e edema agudo, juntamente com outros regimes anti-hipertensivos.^47 - 49^ Uma alternativa para intolerância à nitroglicerina é a administração de bloqueadores dos canais de cálcio di-hidropiridínicos (anlodipino, nicardipina), pois são úteis para pacientes com SCA devido ao seu efeito benéfico sobre o fluxo sanguíneo coronariano. Alternativamente, clevidipina, um bloqueador dos canais de cálcio de curta duração, pode ser administrado por via EV e, por ter um regime de dosagem não baseado no peso, permite uma infusão prolongada e transição bem-sucedida para a terapia oral.^50^ Se disponível, especialmente em SCA com supradesnivelamento do segmento ST, a angioplastia primária é a melhor escolha para a terapia de reperfusão em pacientes com EH, pois a trombólise pode aumentar o risco de sangramento cerebral.^47 - 49 , 51^

Os betabloqueadores, como o labetalol (bloqueador não seletivo dos receptores alfa-1-adrenérgicos), que reduz a resistência vascular sistêmica, mas mantém o FSC, renal e coronariano, ou o esmolol (bloqueador beta-1-cardiosseletivo de início rápido e curta duração de ação) são indicados para atenuar o aumento da frequência cardíaca e reduzir o consumo de oxigênio pelo miocárdio sem comprometer o enchimento diastólico do ventrículo esquerdo e melhorar o prognóstico.^28^ Além disso, a redução da PA diminui o risco de edema pulmonar e o tamanho da zona do infarto.^52^ A tolerância a doses maiores de manutenção do esmolol é um bom preditor de resultados com a terapia oral com betabloqueador.^53^

O valor ótimo de PA após SCA permanece controverso. Numerosos estudos mostraram uma relação inversa entre PAD e eventos adversos cardíacos isquêmicos (ou seja, quanto menor a PAD, maior o risco de doença coronariana e desfechos adversos). Esse efeito é definido como o fenômeno da curva J, que descreve a forma da relação entre a PA e o risco de morbidade e mortalidade cardiovascular.^54^ Esse perfil parece ser mais pronunciado em pacientes com doença arterial coronariana subjacente.^55^

## 9. Disfunção Ventricular Esquerda Aguda na Emergência Hipertensiva

Disfunção ventricular esquerda aguda é mais conhecida pelo termo EAP. A EH, a insuficiência mitral aguda (disfunção do músculo papilar secundária à doença isquêmica ou ruptura espontânea) e a SCA são os fatores causais mais comuns de EAP cardiogênico.^56 , 57^ Cerca de 1/3 dos pacientes admitidos com EAP e EH tem função ventricular esquerda preservada. A EH com quadro de EAP deve ser controlada em UTI, com medicação via parenteral, monitoramento e diminuição gradativa da PA.^58^ Nitroglicerina e nitroprussiato de sódio são utilizados com a finalidade de reduzir a pré e a pós-carga. A administração de diuréticos de alça também diminui sobrecarga de volume e ajuda a reduzir a PA. O uso de pressão positiva contínua de vias aéreas não invasiva pode ajudar ao reduzir edema pulmonar e retorno venoso.^28 , 59^

## 10. Síndromes Aórticas Agudas

Síndrome aórtica aguda (SAA) é o termo atual que abrange a dissecção aórtica (DA), o hematoma intramural (HIM) e as ulcerações ateroscleróticas penetrantes (UAP), com uma incidência que varia de 3,5 a 6,0 por 100.000 pacientes/ano.^60^ Tendo em vista a sua elevada taxa de mortalidade, a SAA deve ser considerada e diagnosticada prontamente em pacientes com dor precordial ou dorsal aguda, principalmente se associada à HAS. Tomografia computadorizada, ressonância magnética e ecocardiografia transesofágica são exames de imagem confiáveis para o diagnóstico de SAA, e a dosagem de D-dímero sérico foi 51,7 a 100% sensível e 32,8 a 89,2% específica em 6 estudos.^61^

A DA é a forma mais comum de SAA, correspondendo a 85 a 95%; HIM acomete de 0 a 25%; e UAP, de 2 a 7%.^61^ De acordo com a classificação de Stanford, as SAA são divididas em tipo A, que envolve a aorta ascendente, e tipo B, que não envolve esse segmento. Já a classificação de DeBakey distingue o tipo I, que envolve pelo menos a aorta ascendente e o arco aórtico e muitas vezes também a aorta descendente; o tipo II, que é confinado à aorta ascendente, e o tipo III, que se origina na aorta descendente distal e acomete a artéria subclávia esquerda.^60^ As SAA podem estar associadas a muitos fatores de risco, como sexo masculino, idade avançada, parente de primeiro grau que tenha sofrido SAA, HAS, dislipidemia, tabagismo, uso de drogas ilícitas, história de grande arterite vascular (p. ex., arterite de Takayasu), doença vascular de colágeno (como síndrome de Marfan, Loeys-Dietz, síndrome de Ehlers-Danlos), trauma fechado em acidente de veículo motorizado ou queda vertical, instrumentação arterial para fins diagnósticos ou terapêuticos ou, ainda, mutações hereditárias em genes para proteínas envolvidas na integridade vascular (como a mutação no gene *ACTA2* ).^60^

### 10.1. Tratamento

O tratamento das SAA requer uma abordagem multidisciplinar envolvendo intervenções clínicas, endovasculares e cirúrgicas.^62^ As DA tipo A têm mau prognóstico e mortalidade intra-hospitalar global de 30% com aumento da mortalidade em 1 a 2% a cada hora de evolução.^63^ Sem intervenção, a mortalidade é de cerca de 58%, em comparação a 26% com a intervenção cirúrgica.^63^ O tratamento cirúrgico aberto é ideal para tratar as SAA de tipo A (aorta ascendente), e o reparo aórtico endovascular torácico é o mais indicado para o tratamento das SAA de tipo B (aorta descendente).^64 - 66^ Nos casos de SAA de tipo B, a mortalidade intra-hospitalar foi significativamente maior após a cirurgia aberta (33,9%) do que após o tratamento endovascular (10,6%, p = 0,002).^66^

O manejo inicial de uma DA envolve o controle de dor e o uso de agentes anti-hipertensivos. Devem-se administrar betabloqueadores por via EV (metoprolol, esmolol ou labetalol) para reduzir o estresse na parede, diminuindo a frequência cardíaca e a PA, mantendo a perfusão cerebral, coronariana e renal adequadas.^60^ A administração de betabloqueadores deve ser completada antes da diminuição da PA com agentes redutores de pós-carga. As diretrizes recomendam uma redução da PAS para 100 a 120 mmHg e uma frequência cardíaca inferior a 60 bpm.^65^ Em caso de intolerância aos betabloqueadores, os bloqueadores dos canais de cálcio não di-hidropiridínicos (verapamil ou diltiazem) devem ser usados.^67^ Após o betabloqueio adequado, deve-se proceder à redução da pós-carga. Embora os inibidores da enzima de conversão da angiotensina (IECA) não tenham demonstrado benefício significativo para a mortalidade, têm sido utilizados como adjuvantes na redução da PA.^68^ O nitroprussiato de sódio também pode ser usado após betabloqueio, pois em monoterapia pode aumentar o estresse de cisalhamento da parede aórtica, resultando em progressão da dissecção.^60^ Não há indicação conhecida para o bloqueio plaquetário agudo no controle da DA até o momento.^60^ Vários estudos mostraram que o uso de estatinas reduz a taxa de crescimento de aneurisma da aorta abdominal (AAA) e também diminui a probabilidade de ruptura recorrente após o reparo.^69^ No entanto, o papel das estatinas nas SAA não está claro.^69^ Deve-se promover o efetivo controle da dor com sulfato de morfina, fentanila ou um opiáceo.^60^

## 11. Emergências Hipertensivas na Gestação

A HAS representa o problema médico mais comum na gestação, manifestando-se em até 10% das gestações e respondendo por cerca de 25% das admissões hospitalares pré-natais, além de ser importante causa de morbidade e mortalidade materna e fetal. Mulheres que tiveram HAS na gestação apresentam maior risco para doença hipertensiva, AVC e doença arterial coronariana no futuro.^70 , 71^ Para definição de HAS na gravidez, adota o mesmo critério das Diretrizes de Hipertensão, ou seja, PA ≥ 140/90 mmHg. Considera-se HAS grave na gestação quando os valores da PAS ≥ 160 a 170 mmHg e a PAD ≥ 110 mmHg.^72^ Assim, HAS pode preceder (no caso, HAS crônica) ou se desenvolver durante o curso da gestação (pré-eclâmpsia/eclâmpsia/hipertensão gestacional), caracterizando quatro diferentes categorias de HAS:^70 - 72^

1. Hipertensão crônica tem início antes da gravidez ou é diagnosticada antes da 20^a^ semana de gestação. Somente 20 a 25% dos casos de HAS crônica da gravidez evoluem para pré-eclâmpsia.
2. Hipertensão gestacional é o distúrbio mais comum (10% dos casos ocorrem nas primigestas; 20 a 25% dos casos se apresentam sobrepostos à HAS crônica). Aparece após a 20^a^ semana e não se acompanha de proteinúria. A PA retorna a valores normais 1 a 2 semanas após o parto. Cursa com bom prognóstico materno e fetal.
3. Pré-eclâmpsia / eclâmpsia. Pré-eclâmpsia é um processo específico da gravidez, definido por HAS que aparece após a 20^a^ semana de gestação, cursa com proteinúria (> 300 mg/24 h ou relação proteína/creatinina > 300 mg/g), edema, e às vezes, alterações da coagulação e da função hepática. A pré-eclâmpsia pode progredir rapidamente para eclâmpsia, condição clínica caracterizada por convulsões tônico-clônicas precedida de hipertensão grave, cefaleia e hiperreflexia. Hemorragia cerebral é a complicação mais grave com elevado índice de mortalidade materna. Proteinúria e valores pressóricos elevados devem retornar para valores normais em até 12 semanas após o parto.
4. Hipertensão crônica com pré-eclâmpsia/eclâmpsia sobreposta. Essa suspeita deve ser lembrada quando surgir microalbuminúria (30 a 300 mg/urina de 24 h ou 30 a 300 mg/g na relação albumina/creatinina em amostra de urina isolada) ou aumento de proteinúria pré-existente, alteração clínica ou laboratorial característica de pré-eclâmpsia ou elevação dos níveis de PA pré-existentes após a 20^a^ semana de gestação em portadora de HAS crônica.

### 11.1. Tratamento

Os 2 principais pontos-chave no tratamento da CH na gestação são: (1) estabilização da mãe, incluindo o uso de anti-hipertensivos seguros e apropriados para uso na gravidez e indicação do parto e (2) bem-estar fetal, que deve ser confirmado por monitoramento fetal e ultrassonografia.

O tratamento farmacológico deve ser iniciado quando a PA estiver > 150/100 mmHg, com o objetivo de mantê-la em 130 a 150/80 a 100 mmHg (grau de recomendação [GR]: IIa; nível de evidência [NE]: B). Em pacientes com pré-eclâmpsia, com quadro clínico estabilizado sem necessidade de parto imediato, é indicado tratamento anti-hipertensivo oral.^72^ No Brasil, os medicamentos orais usualmente empregados são a metildopa, hidralazina, antagonistas dos canais de cálcio (nifedipina de ação prolongada, anlodipino) e betabloqueadores (de preferência, pindolol). Os tiazídicos podem ser continuados em gestantes com HAS crônica, desde que não promovam depleção de volume.^73^ O uso de bloqueadores do sistema renina-angiotensina é contraindicado na gestação (GR: I; NE: B).^72^

O tratamento medicamentoso urgente é indicado em HAS grave (PAS > 155 a 160 mmHg) e na presença de sinais premonitórios (GR: I; NE: B). Usa-se hidralazina por via EV (5 mg, repetir 5 a 10 mg a cada 30 minutos até o máximo de 20 mg). Nitroprussiato de sódio pode ser considerado para controle urgente da PA, especialmente, na presença de EAP e HAS grave e refratária.^72^

O sulfato de magnésio é o fármaco de escolha tanto para o tratamento como para prevenção das crises convulsivas durante a eclâmpsia. A paciente deve ser monitorada em relação a débito urinário, reflexos patelares, frequência respiratória e saturação de oxigênio. O magnésio plasmático deve ser mantido entre 4 e 7 mEq/L e deve ser dosado na presença de doença renal. Na suspeita de intoxicação por sulfato de magnésio, usar gluconato de cálcio.^70 , 71^

## 12. Emergências Adrenérgicas

**Figura 4:**
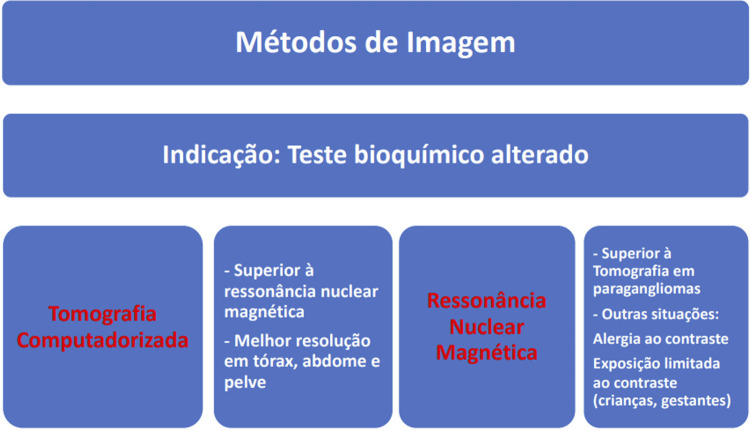
– *Métodos de imagem utilizados para confirmação diagnóstica de feocromocitoma.*

Tumores neuroendócrinos associados ao tecido simpático que têm a potencialidade de secretar catecolaminas são raros e chamados de feocromocitomas (medula adrenal) ou paragangliomas (tecido não adrenal). Diagnóstico, localização e delineação anatômica destes tumores envolvem dosagem das catecolaminas e seus metabólitos no sangue e na urina, tomografia computadorizada e/ou ressonância magnética e cintilografia com metaiodobenzilguanidina (I^123^). A sintomatologia pode ocorrer em qualquer fase da vida e não é específica, dependendo da forma de liberação das catecolaminas no sangue, podendo ocorrer elevação da PA, palpitações e cefaleia. A remoção cirúrgica destes tumores sempre é indicada para curar ou prevenir a doença cardiovascular secundária ao excesso de catecolaminas.^74^ A elevação pressórica nestes pacientes pode ser mantida ou paradoxal, e aumento acentuado da PA pode caracterizar EH com risco iminente de vida. Isso ocorre por ativação dos alfarreceptores pelas catecolaminas. A Diretriz Brasileira de Hipertensão Arterial sugere um fluxograma de abordagem diagnóstica para os tumores neuroendócrinos (feocromocitoma e paragangliomas), que é mostrado na Tabela 4 .^75^ A Figura 4 mostra os métodos de imagem utilizados para confirmação diagnóstica diante de um teste bioquímico alterado.

**Tabela 4:** – Fluxograma da VII Diretriz de Hipertensão Arterial para diagnóstico clínico e laboratorial em casos de feocromocitoma e paraganglioma

Achados clínicos	Suspeita diagnóstica	Estudos adicionais
- Hipertensão paroxística com cefaleia, sudorese e palpitações	Feocromocitoma	- Metanefrinas plasmáticas livres
- Hipertensão resistente		- Metanefrinas urinárias e catecolaminas séricas
		- Exames de imagem

A cintilografia de corpo inteiro visa localizar tumores neuroendócrinos extra-adrenais (paragangliomas). É indicada quando o teste bioquímico estiver alterado e os exames de imagens forem negativos. Sempre deve ser realizada após verificação e suspensão de fármacos que possam interferir com a sua interpretação (simpatomiméticos, bloqueadores dos canais de cálcio, cocaína, antidepressivos e labetalol), devendo suspendê-los por 14 dias prévios ao exame. É contraindicada na gestação.^76^ Diagnosticado o tumor neuroendócrino, o tratamento proposto é sempre cirúrgico, precedido por preparo farmacológico e hidratação, com o objetivo de prevenir ou atenuar CHs ou hipotensão durante o ato operatório ( Tabela 5 ).^76^ Nessa situação, são administrados fármacos anti-hipertensivos por via EV (inicialmente, alfabloqueadores, e posteriormente, betabloqueadores). Nitroprussiato de sódio em infusão contínua (0,25 a 10 mg/kg/min) ou fentolamina (infusão contínua 1 a 5 mg com dose máxima de 15 mg) podem ser utilizados, se houver elevação mais acentuada da PA.^75 - 77^

**Tabela 5:** – Cuidados pré-operatórios em casos de feocromocitoma

Dieta hipersódica e hidratação (faltam evidências):
- Infusão salina no ato operatório (1 a 2 L)
- Reverter contração de volume
- Prevenir hipotensão

**Preparo farmacológico:**

- Bloqueio alfa-adrenérgico
- Betabloqueadores
- Bloqueadores dos canais de cálcio
- Não existem evidências quanto à meta pressórica

**Adrenalectomia via laparoscópica (maioria dos casos):**

- Para paragangliomas (minoria)

**Adrenalectomia a céu aberto (para paragangliomas):**

- Para feocromocitoma (minoria)

## 13. Drogas Ilícitas e Emergência Hipertensiva

Na sala de emergência, pacientes que se apresentam com CH e hiperatividade simpática levantam a suspeita de intoxicação por anfetamina ou cocaína, assim como uso abusivo de outros fármacos, tais como inibidores da recaptação da serotonina, inibidores da monoaminoxidase e uso de drogas citotóxicas ou antiangiogênicas.^52^

A cocaína tem múltiplos efeitos cardiovasculares e hematológicos que contribuem para elevação da PA, desenvolvimento de isquemia miocárdica e/ou IAM por vasoconstrição coronariana. A cocaína, mesmo em pequenas doses, bloqueia a recaptação de norepinefrina e dopamina nos terminais adrenérgicos pré-sinápticos, causando acúmulo de catecolaminas no receptor pós-sináptico, atuando, assim, como um poderoso agente simpatomimético.^78^ Consequentemente causa aumento da frequência cardíaca e da PA de maneira dose-dependente.^79^ Além disso, o consumo de cocaína pode reduzir a função ventricular esquerda associada a aumento do estresse parietal no final da sístole e ampliação da demanda de oxigênio. Os efeitos cronotrópicos do uso de cocaína são intensificados pelo consumo de álcool.^80^ A vasoconstrição induzida pelo uso de cocaína é secundária à estimulação dos receptores alfa-adrenérgicos nas células musculares lisas da circulação coronariana. Ainda, tal droga aumenta a liberação de endotelina-1^81^ e diminui a biodisponibilidade de óxido nítrico, promovendo elevação da PA.^82^ O tratamento com benzodiazepínicos tem indicação inicial. Quando a redução da PA for necessária, um fármaco alfabloqueador competitivo por via EV será indicado (fentolamina). Alternativamente, nicardipina ou nitroprussiato de sódio poderão ser considerados.^83^ Clonidina também pode ser considerada, pois, além da sua ação simpatolítica, tem efeito sedativo.

Nas SCA, o tratamento com nitroglicerina e aspirina é recomendado concomitantemente com benzodiazepínicos. Na presença de SCA com taquiarritmias, os bloqueadores dos canais de cálcio não di-hidropiridínicos (diltiazem e verapamil) são recomendados. Os betabloqueadores (incluindo labetalol) são contraindicados, pois não reduzem a vasoconstrição coronariana.^84^ A nicardipina também pode ser uma boa alternativa para pacientes com EH induzida por drogas citotóxicas ou antiangiogênicas.

## 14. Emergência Hipertensiva no Pós-Operatório de Cirurgia Vascular

O conceito de “emergência hipertensiva pós-operatória” difere da emergência/urgência hipertensiva ambulatorial, em virtude da ocorrência dessa situação clínica única em um ambiente atípico (pós-operatório). Notadamente, valores de PA moderadamente elevados no contexto pós-operatório podem requerer tratamento imediato.^85^

Emergência hipertensiva pós-operatória (EHPO) é arbitrariamente definida como elevação da PAS > 190 mmHg e/ou PAD > 100 mmHg, confirmada em duas leituras consecutivas durante o período pós-operatório imediato.^86^ Elevação da PAS em 40 a 50 mmHg ou incremento dos valores pressóricos superiores a 20% em relação aos valores basais também podem caracterizar hipertensão pós-operatória.^87^ Esse aumento dos valores pressóricos geralmente começa 10 a 20 minutos após a cirurgia e pode durar até 4 horas. A fisiopatologia da EHPO em pacientes previamente normotensos está associada a vasoconstrição periférica, liberação de catecolaminas, redução da sensibilidade dos barorreceptores, ativação adrenérgica central, liberação de vasopressina, estimulação do sistema renina-angiotensina com consequente produção de angiotensina II, liberação de citocinas inflamatórias (IL-6) e retenção de sódio. Todas essas alterações resultam em vasoconstrição, aumento da pós-carga, elevação da PAS/PAD e taquicardia. Se não tratada, a hipertensão pós-operatória aumenta o risco de isquemia miocárdica, IAM, EAP, AVC, sangramento e mortalidade pós-operatória.^88 , 89^

EHPO ocorre em 40 a 80% dos pacientes submetidos à endarterectomia carotídea ou cirurgia cardíaca aberta, em 57% dos pacientes submetidos à cirurgia de aorta abdominal e 29% dos pacientes submetidos à cirurgia vascular periférica.^90 - 92^ Especialmente, HAS aguda e grave com elevação da PAS > 220 mmHg pode ocorrer em 9% dos indivíduos submetidos à endarterectomia carotídea.^93^ Essa manifestação, que pode ser transitória, está relacionada à manipulação do seio carotídeo e pode provocar hematoma, isquemia miocárdica e hiperperfusão cerebral com consequente lesão neurológica.^94^ Outros mecanismos propostos incluem denervação iatrogênica,^95^ diminuição da atividade do barorreflexo,^96^ redução da sensibilidade do seio carotídeo e aumento da produção de renina cerebral e/ou de catecolaminas.^97 , 98^

EH também pode ocorrer após correção cirúrgica da coarctação da aorta. A etiologia é multifatorial, como alteração do reflexo barorreceptor, ativação do sistema simpático e do sistema renina-angiotensina e expansão do volume extracelular.^99^ A estimulação das fibras nervosas simpáticas localizadas nas camadas média e adventícia do istmo aórtico têm dois efeitos, ambos resultando em hipertensão. Inicialmente, ocorre liberação periférica de norepinefrina com consequente vasoconstrição e elevação da PA. A seguir, pode ocorrer estimulação das células justaglomerulares que liberam renina e promovem hipertensão adicional. Secundariamente, esse aumento da produção de renina provoca desvio de sangue das artérias mesentéricas, causando assim os sintomas abdominais da chamada síndrome pós-coarctectomia.^100^

Antes de iniciar o tratamento farmacológico anti-hipertensivo, causas reversíveis de hipertensão pós-operatória devem ser investigadas, tais como: dor, hipoxia, hipercarbia, agitação, distensão vesical e hipervolemia.^101^ Analgesia apropriada e sedação são consideradas requisitos antes de iniciar a terapia anti-hipertensiva.^102^ Quando EHPO estiver presente, a distinção entre emergência e urgência será mandatória.^1 - 4^ O objetivo do controle é interromper a lesão vascular e reverter o processo patológico, e não necessariamente normalizar a PA. Reduções progressivas da PA, conforme relatadas nos princípios gerais do tratamento das EH, devem ser obtidas.^1^
