# Supplementary material for: Posicionamento Luso-Brasileiro de Emergências Hipertensivas – 2020
Source: Arq Bras Cardiol. 2020 May 12;114(4):736–51. [Article in Portuguese] doi: 10.36660/abc.20190731 (PMC9744343; doi:10.36660/abc.20190731)
Supplement: Translation

# Translation: Luso-Brazilian Position Statement on Hypertensive Emergencies – 2020

**Authors:** José Fernando Vilela-Martin, Juan Carlos Yugar-Toledo, Manuel de Carvalho Rodrigues, Weimar Kunz Sebba Barroso, Luís Carlos Bronze S. Carvalho, Francisco José Torres González, Celso Amodeo, Vitor Manuel Margarido Paixão Dias, Fernando Carvalho Moreira Pinto, Luís Filipe Reis Martins, Marcus Vinícius Bolívar Malachias, Paulo Cesar Veiga Jardim, Dilma do Socorro Moraes de Souza, Oswaldo Passarelli, Eduardo Costa Duarte Barbosa, Jorge Junqueira Polonia, Rui Manoel dos Santos Póvoa

**Affiliations:** 1 Brazil Faculdade Estadual de Medicina de São José do Rio Preto (FAMERP), São José do Rio Preto, SP – Brazil; 2 Portugal Centro Hospitalar Universitário Cova da Beira, Covilhã – Portugal; 3 Portugal Universidade da Beira Interior, Covilhã – Portugal; 4 Brazil Liga de Hipertensão Arterial da Universidade Federal de Goiás (UFG), Goiânia, GO – Brazil; 5 Portugal Marinha Portuguesa, Lisboa – Portugal; 6 Espanha Vithas Hospital Nisa 9 de Octubre, Valencia – Espanha; 7 Brazil Universidade Federal de São Paulo (UNIFESP), São Paulo, SP – Brazil; 8 Portugal Centro Hospitalar de Vila Nova Gaia, Espinho – Portugal; 9 Portugal Centro Hospitalar de Entre o Douro e Vouga, E.P.E., Santa Maria da Feira – Portugal; 10 Portugal Hospital Escola da Universidade Fernando Pessoa, Cosme – Portugal; 11 Brazil Faculdade de Ciências Médicas de Minas Gerais, Belo Horizonte, MG – Brazil; 12 Brazil Universidade Federal de Goiás (UFG), Goiânia, GO – Brazil; 13 Brazil Hospital do Coração De Goiás, Goiânia, GO – Brazil; 14 Brazil Faculdade de Medicina da Universidade Federal do Pará (UFPA), Belém, PA – Brazil; 15 Brazil Instituto Dante Pazzanese de Cardiologia, São Paulo, SP – Brazil; 16 Brazil Liga de Combate à Hipertensão de Porto Alegre, Porto Alegre, RS – Brazil; 17 Portugal Faculdade de Medicina da Universidade do Porto, Porto – Portugal

**Table:** 

Declaration of potential conflict of interests of authors/collaborators of the Luso-Brazilian Position Statement on Hypertensive Emergencies – 2020 If, within the last 3 years, the author/collaborator of the statement:							
Names of statement collaborators	Participated in clinical and/or experimental studies sponsored by pharmaceutical or equipment companies related to this guideline	Spoke at events or activities sponsored by industry related to this guideline	Was (is) a member of a board of advisors or a board of directors of a pharmaceutical or equipment industry	Participated in normative committees of scientific research sponsored by industry	Received personal or institutional funding from industry	Wrote scientific papers in journals sponsored by industry	Owns stocks in industry
Celso Amodeo	Medtronic	No	No	No	Novonordisk, Pfizer, Sankyo	Medley	No
Dilma do Socorro Moraes de Souza	No	No	No	No	No	No	No
Eduardo Costa Duarte Barbosa	No	Servier, EMS	No	No	Servier, EMS, Torrent	EMS, Medley, Novartis	No
Fernando Carvalho Moreira Pinto	No	No	No	No	No	No	No
Francisco José Torres González	No	No	No	No	No	No	No
Jorge Junqueira Polonia	No	No	No	No	No	No	No
Jose Fernando Vilela-Martin	No	No	No	No	No	No	No
Juan Carlos Yugar-Toledo	No	No	No	No	No	No	No
Luís Carlos Bronze S. Carvalho	No	No	No	No	No	No	No
Luís Filipe Reis Martins	No	No	No	No	No	No	No
Manuel de Carvalho Rodrigues	No	No	No	No	No	No	No
Marcus Vinícius Bolivar Malachias	No	Libbs, Biolab	No	No	No	Libbs, Biolab, Aché	No
Oswaldo Passarelli Júnior	No	No	No	No	No	No	No
Paulo César Veiga Jardim	No	No	No	No	No	Biolab, Aché, Libbs	No
Rui Manoel dos Santos Póvoa	No	No	No	No	No	No	No
Vitor Manuel Margarido Paixão Dias	No	No	No	No	Servier, Tecnimede	No	No
Weimar Kunz Sebba Barroso	Boehringer, Torrent, EMS, Amgen, AstraZeneca, Novartis	EMS, Servier, Medley, Omron, Cardios	Omron	No	EMS, Servier	EMS, Servier, Medley	No

## Content

1. Definition, Epidemiology, and Classification of Hypertensive Emergencies 738

2. Pathophysiological Aspects of Hypertensive Emergency 739

2.1. Autoregulation of Cerebral Blood Flow 739

3. Clinical and Laboratory Assessment 740

4. Treatment of Hypertensive Emergencies: General Principles, Main Medications and Dosages 740

5. Hypertensive Encephalopathy 741

5.1. Clinical Manifestations 741

5.2. Diagnosis 741

5.3. Treatment 741

6. Malignant or Accelerated Hypertension 742

7. Stroke and Hypertensive Emergency 742

7.1. Ischemic Stroke 743

7.2. Hemorrhagic Stroke 744

8. Acute Coronary Syndromes and Hypertensive Emergency 744

9. Acute Left Ventricular Dysfunction in Hypertensive Emergency 744

10. Acute Aortic Syndromes 745

10.1. Treatment 745

11. Hypertensive Emergencies During Pregnancy 745

11.1. Treatment 746

12. Adrenergic Emergencies 746

13. Illicit Drugs and Hypertensive Emergency 746

14. Postoperative Hypertensive Emergency Following Vascular Surgery 747

References 748

## 1. Definition, Epidemiology, and Classification of Hypertensive Emergencies

Hypertensive emergencies (HEs) comprise a wider nosological condition known as hypertensive crisis (HC). HC represents clinical situations with acute blood pressure (BP) elevation, often with levels of systolic BP (SBP) ≥ 180 mmHg and diastolic BP (DBP) ≥ 120 mmHg, which may or may not result in **target-organ damage** (TOD) (heart, brain, kidneys, and arteries).^1 - 5^ HCs may present in two distinct forms in relation to severity and prognosis: hypertensive urgency (HU) and HE. Cases of HE have a marked elevation in BP associated with TOD and immediate risk of death, a fact that requires a rapid and gradual reduction in BP levels within minutes to hours, with intensive monitoring and use of intravenous medications.^1 - 5^ HEs can manifest as cardiovascular, cerebrovascular, or renal events or as a pregnancy-related event in the form of preeclampsia or eclampsia. Although the classic definition of both HC presentations describes this condition with values above 180/120 mmHg, the largest current consensus is established on the concept that what distinguishes HEs from HUs is, more than the BP value, the occurrence of damage or imminent risk of target-organ involvement. Thus, HUs are characterized by BP elevations without TOD or imminent risk of death, a fact that allows for a slower reduction in BP levels over a period of 24 to 48 hours. Currently, there is a wide discussion about the actual existence of the diagnosis of “hypertensive urgency.”^6^ Many advocate that this classification needs to be updated (if not abandoned) and that, instead of the BP value, the main diagnostic importance lies in the observation of signs/symptoms and acute TOD. Others believe that the correct term should be “BP elevation without evolving TOD.”^5 , 7^

As discussed, even though the BP levels are often very high (≥ 180/120 mmHg), HEs are defined by TOD and not by BP levels. Therefore, the numerical pattern that defines HC is conceptual and serves as a therapeutic parameter, but should not be used as an absolute criterion.

If the definition of HC is more universally accepted today, the knowledge about the epidemiology and prevalence of this condition by the scientific community is still limited. The literature has only a few studies on the subject, all of which conducted in a small number of participants. Non-adherence to treatment is currently hypothesized to be one of the most prevalent factors in the etiology of HC, without distinction between HU and HE. The incidence of HC in the largest serial studies in the US was about 4.8%, with 0.8% attributed to HEs.^8 , 9^ Other centers have shown that HCs account for a variable rate of 0.45 to 0.59% of all hospital emergency care and 1.7% of all clinical emergencies, with HU being more common than HE.^10 - 12^ Ischemic stroke and acute pulmonary edema (APE) are the most common clinical conditions in HE.^10 , 11^ Estimates indicate that about 1% of all hypertensive individuals will probably develop an episode of HC over their lifetimes.^1 , 2^ The clinical conditions with TOD implicated in HEs are shown in Table 1 . Table 2 shows the main conditions associated with HUs.

**Table 1:** – Conditions with target-organ damage characterizing hypertensive emergencies1-5

Severe hypertension associated with acute complications
**Cerebrovascular events**
- Hypertensive encephalopathy
- Intracerebral hemorrhage
- Subarachnoid hemorrhage
- Ischemic stroke
**Cardiocirculatory events**
- Acute aortic dissection
- Acute pulmonary edema with left ventricular failure
- Acute myocardial infarction
- Unstable angina
**Renal disease**
- Rapidly progressive renal failure

**Severe adrenergic crisis**

- Pheochromocytoma crisis
- Illicit drug overdose (cocaine, crack, LSD)

**Hypertension in pregnancy**

- Eclampsia
- Severe preeclampsia
- "HELLP" syndrome
- Severe hypertension in late pregnancy

*HELLP: hemolysis, elevated liver enzymes, and low platelet count; LSD: lysergic acid diethylamide.*

**Table 2:** – Conditions associated with hypertensive urgency1-5

Severe hypertension associated with:
- Coronary insufficiency
- Cardiac insufficiency
- Aortic aneurysm
- Uncomplicated stroke
- Severe epistaxis
- Extensive burns
- Hypocoagulability states

**Systemic vasculitis**

- Perioperative
- Preoperative in emergency surgeries
- Intraoperative (cardiac surgery, vascular surgery, neurosurgery, pheochromocytoma, etc.)
- Postoperative stage III hypertension (organ transplantation, cardiac surgery, vascular surgery, neurosurgery, etc.)

**Mild/moderate adrenergic crisis**

- Rebound syndrome (abrupt discontinuation of adrenergic inhibitors)
- Drug-food interaction (tyramine vs. MAO inhibitors)
- Excessive use of stimulants (amphetamines, tricyclics, etc.)

**In pregnancy**

- Preeclampsia
- Stage III hypertension

*MAO: monoamine oxidase.*

## 2. Pathophysiological Aspects of Hypertensive Emergency

The pathophysiology of HE has not been completely elucidated, and in general, two different mechanisms may play central roles in this process. The first is an imbalance in the vascular autoregulation system leading to reduced perfusion pressure and, consequently, decreased blood flow and increased vascular resistance, resulting in mechanical stress and endothelial injury.^13^ The second mechanism is an activation of the renin-angiotensin system resulting in greater vasoconstriction and leading to a vicious cycle of endothelial injury, fibrinoid necrosis of arterioles, and subsequent ischemia.^14^ Vascular injury leads to platelet and fibrin deposition, also characterizing a prothrombotic state.^15^ Subsequent ischemia results in the release of more vasoactive substances, creating a vicious cycle.

### 2.1. Autoregulation of Cerebral Blood Flow

Knowledge about the mechanism of autoregulation of blood flow to target organs (brain, coronary arteries, and kidneys) is fundamental for improved antihypertensive treatment in cases of HE. Autoregulation of cerebral blood flow (CBF) is maintained by the ratio of cerebral perfusion pressure (CPP) to cerebrovascular resistance (CVR), *i.e.* , CBF = CPP/CVR (CPP = mean BP - mean venous pressure). CPP is the difference between BP – which helps with tissue blood flow – and venous pressure. With a normal CPP, venous pressure is not important, so CPP is equivalent to BP. Reductions in CPP may be caused by reductions in BP or increased intracranial pressure (ICP), which increases venous pressure. Elevations in ICP may occur as a result of arterial or venous occlusive disease or intracerebral hemorrhage. In normotensive individuals, a wide variation in BP (between 60 and 150 mmHg) may occur without CBF changes. An increase in CPP (or BP) leads to an elevation in CVR, thus protecting the patient against cerebral edema, while reductions in CPP result in decreased CVR, thus protecting the patient from tissue ischemia. When CPP exceeds the upper limit of autoregulation, CBF increases, causing cerebral edema. In contrast, when CPP falls below the lower limit of autoregulation, CBF decreases, causing cerebral ischemia.^16 , 17^

In hypertensive individuals, this relationship is modified in a way that their lower limit of autoregulation is higher compared with normotensive individuals. Thus, improper decrease in CPP can hinder tissue irrigation and, consequently, aggravate the viable ischemic area. For this reason, it is advisable to initially reduce the mean BP by 20 to 25% in relation to the initial values, as this will bring them close to the lower autoregulation limit.^18^ Attention should be given to this situation, as most patients with HE have chronic hypertension with the pressure/flow (cerebral, coronary, and renal) autoregulation curve shifted to the right and do not present acute TOD, which is why a sudden decrease in BP may be associated with significant morbidity.^18 - 20^

## 3. Clinical and Laboratory Assessment

When managing a HE, the practitioner should discriminate between emergency and urgency, establishing a correct diagnosis of the various HE situations in order to select the most appropriate therapy for each TOD. This is very important since the correct diagnosis and treatment may prevent worsening of the clinical condition due to the critical situation. The approach to patients with HE requires clinical evaluation and complementary tests performed in clinical emergency centers with hospital support. BP should be measured in both arms (at least three measurements), preferably in a quiet environment. Individuals with acute BP elevations often present metabolic abnormalities characterized by hyperglycemia, dyslipidemia, lower potassium levels, and reduced renal function.^21^ The sequence of steps in the management of patients with HC is as follows:^1 - 5 , 22 , 23^

1. Seek factors that may have triggered the acute BP elevation.
2. Investigate symptoms or situations that simulate HC (headache, labyrinthitis, physical trauma, pain, emotional stress, and family or professional problems).
3. Observe history and duration of hypertension, use of antihypertensive drugs (doses and pharmacological adherence).
4. Investigate prior episodes similar to the current situation.
5. Investigate the use of medications that may interfere with BP control (anti-inflammatory drugs, steroids, analgesics, antidepressants, appetite suppressants).
6. Evaluate the use or abuse of alcohol and toxic substances (cocaine, crack, lysergic acid diethylamide [LSD]).
7. Investigate the use of suddenly discontinued adrenergic inhibitors (clonidine, methyldopa, and beta-blockers).
8. Observe the association with other morbidities and risk factors (diabetes, cardiac disease, renal disease, smoking, dyslipidemia).
9. Clinical history and physical examination should be performed according to the presence of TOD:

- Central nervous system (observe the occurrence of headache, dizziness, visual and speech disorders, consciousness level, agitation or apathy, confusion, focal neurological deficits, neck stiffness, seizure, and coma).
- Cardiovascular system (assess heart rate, symptoms of palpitations, and presence of carotid murmur; investigate the occurrence of thoracic, precordial, abdominal, and back pain and discomfort, in addition to signs and symptoms of left ventricular failure including gallop rhythm, dyspnea, jugular venous stasis, peripheral pulses, and oxygen saturation).
- Renal and genitourinary system (assess changes in urinary volume, frequency, and characteristics, dehydration, lower limb edema, hematuria, and dysuria). Note: examination of the abdomen (for pulsatile abdominal masses and abdominal murmur) should not be overlooked.
- Fundoscopy (observe the occurrence of vasospasm, arteriovenous nicking, arteriolar wall thickening and aspect of copper or silver-wiring, hard and soft exudates, hemorrhages, and papilledema).

Complementary tests should be performed according to the involvement of target organs:

- Central nervous system (computed tomography, magnetic resonance imaging, and lumbar puncture).
- Cardiovascular system (electrocardiography, chest x-ray, echocardiography, markers of myocardial necrosis, angiotomography, magnetic resonance imaging).
- Renal system (urinalysis, urea, creatinine, electrolytes, and blood gases).

## 4. Treatment of Hypertensive Emergencies: General Principles, Main Medications and Dosages

Better diagnostic and therapeutic conditions have led to a great reduction in 1-year mortality, which improved from 80% in 1928 and 50% in 1955 to only 10% in 1989.^24 , 25^ The aim of treating patients with clinical manifestations of HE is to reduce BP rapidly to prevent the progression of TOD. Patients should be admitted to an intensive care unit, undergo intravenous antihypertensive treatment, and be carefully monitored during parenteral therapy to prevent the occurrence of hypotension. The general recommendations for BP reduction suggested by the Seventh Report of the Joint National Committee (JNC)^26^ for HEs are summarized as follows:

- ↓ BP ≤ 25% within the first hour.
- ↓ BP 160/100 to 110 mmHg in 2 to 6 hours.
- BP 135/85 mmHg at 24 to 48 hours.

However, HEs should be addressed considering the affected system or target organ. Thus, each type of HE (cardiovascular, cerebral, renal, and others) should be characterized prior to starting specific antihypertensive therapy (see “Clinical and Laboratory Evaluation”).

Several pharmacological therapies are currently available for HE treatment. The ideal antihypertensive medication for parenteral use must present the following characteristics: ability to reverse the involved pathophysiological abnormalities, rapid onset of action, predictable dose-response curve, minimal dose adjustment, high selectivity, no increase in ICP, prompt reversibility, low risk of promoting hypotension, easy substitution for oral medications, and satisfactory cost-benefit ratio. Table 3 summarizes the pharmacokinetic and pharmacodynamic properties of the main antihypertensive medications used in HE.^2 , 22 , 26 - 28^ In Brazil, the following medications are available for use in HEs: sodium nitroprusside, nitroglycerin, labetalol, esmolol, metoprolol, hydralazine, and enalaprilat.

**Table 3:** – Pharmacokinetic and pharmacodynamic properties of the main antihypertensive medications for parenteral use

Medications	Method of administration and dosage	Start	Duration	Advantages	Disadvantages
Nitroglycerin (nitric oxide donor with arterial and venous vasodilation effects)	Continuous infusion 5 to 15 mg/h	2 to 5 min	3 to 5 min	Coronary perfusion	Headache, variable efficacy, tachyphylaxis
Sodium nitroprusside (arterial and venous vasodilator)	Continuous infusion 0.5 to 10 μg/kg/min	Immediate	1 to 2 min	Titration	Intoxication by thiocyanate, hypotension, nausea, vomiting, muscle spasm
Metoprolol (beta-blocker)	Loading dose: 5 mg IV (repeat every 10 min, up to 20 mg if necessary)	5 to 10 min	3 to 4 h	Reduction in O_2_consumption	Bradycardia, AVB, bronchospasm
Labetalol (alpha- and beta-blocker)	Loading dose: 20 to 80 mg every 10 min Continuous infusion 2 mg/min (maximum 300 mg/24 h)	5 to 10 min	2 to 6 h	Beta-blocker and vasodilator	Nausea, vomiting, AVB, bronchospasm, orthostatic hypotension
Esmolol (Ultra-fast action, ultra-selective beta-blocker)	Loading dose: 500 μg/kg Intermittent infusion: 25 to 50 μg/kg/min ↑ 25 μg/kg/min every 10 to 20 min. Maximum: 300 μg/kg/min	1 to 2 min	1 to 20 min	Selective beta-blocker	Bradycardia, AVB, bronchospasm
Hydralazine (direct-acting vasodilator)	10 to 20 mg IV or 10 to 40 mg IM every 6 h	10 to 20 min IV or 20 to 30 min IM	3 to 12 h	Eclampsia or impending eclampsia	Tachycardia, headache, vomiting. Worsening of angina and AMI. Beware of increased intracranial pressure
Enalaprilat (ACEI)	Intermittent infusion: 1.25 to 5 mg every 6 h	15 min	4 to 6 h	CHF, acute LVF	Hypotension, renal insufficiency
Furosemide (loop diuretic)	Infusion	5 to 10 min	30 to 90 min	CHF, LVF	Hypokalemia

*AMI: acute myocardial infarction; CHF: congestive heart failure; LVF: left ventricular failure; AVB: atrioventricular block; ACEI: angiotensin-converting enzyme inhibitor; IV: intravenous; IM: intramuscular.*

## 5. Hypertensive Encephalopathy

Hypertensive encephalopathy is a neurological dysfunction defined by signs and/or symptoms of cerebral edema secondary to sudden and/or sustained BP elevation. It occurs in individuals with chronic hypertension who develop malignant hypertension or in those previously normotensive who may present acute BP elevations due to other mechanisms, progressing with failure in mechanisms of cerebral perfusion autoregulation. Hypertensive encephalopathy is a diagnosis of exclusion confirmed retrospectively when the neurological condition improves after BP control.

### 5.1. Clinical Manifestations

Hypertensive encephalopathy may present with the insidious onset of holocranial headache, nausea, or vomiting. Subsequently, changes in mental status and visual field, photopsia, blurred vision, visual hallucinations, generalized seizures, hyperreflexia, and signs of intracranial hypertension may develop.^29 , 30^ By the time the neurological manifestations emerge, the DBP is usually above 125 mmHg. The resolution of this condition, from both clinical and imaging standpoints, occurs on average several weeks after BP control. The occurrence of a persistent deficit is a sign of focal neurological injury.

### 5.2. Diagnosis

Magnetic resonance imaging is the most valuable diagnostic test. T2-weighted sequences show hyperintense white matter lesions with preferential involvement of the parieto-occipital regions. The territory irrigated by the vertebrobasilar system can be compromised in more severe cases. Hyperintense signal in apparent diffusion coefficient allows for the visualization of vasogenic edema.^31^ Laboratory tests may show thrombocytopenia, microangiopathic hemolytic anemia, proteinuria, and increased plasma creatinine and liver enzymes. On computed tomography, focal or diffuse hypodensities in the white matter and cortex are common, along with signs of edema. Electroencephalography shows generalized slowing with loss of alpha rhythm, or epileptiform activity if seizures occur.

### 5.3. Treatment

The goal is to reduce the average BP by approximately 10 to 15% in the first hour and by no more than 25% at the end of the first day of treatment. Greater and faster decreases may lead to cerebral hypoperfusion and loss of vascular autoregulation mechanisms.^32 , 33^ Due to the need for rapid BP control, intravenous medications are recommended, of which the most frequently used are sodium nitroprusside (arterial and venous vasodilator), nicardipine (dihydropyridine calcium-channel blocker with arteriolar vasodilation action), clevidipine (short-acting dihydropyridine calcium-channel blocker), labetalol (alpha-adrenergic and beta-adrenergic blocker), or fenoldopam (peripheral dopamine-1 receptor agonist). During pregnancy, magnesium sulfate, diazoxide, or hydralazine are recommended. Corticosteroids (dexamethasone), mannitol (may be used in the absence of renal disease), and anticonvulsants (in case of seizures) may also be used.^23 , 30^ Within the first 24 to 48 hours, oral medications should be introduced to improve BP control (renin-angiotensin-aldosterone system blockers and calcium-channel blockers), with a gradual DBP reduction to values below 90 mmHg in the following 2 to 3 months.^1 , 2 , 5 , 22^

## 6. Malignant or Accelerated Hypertension

Malignant hypertension is characterized by hypertension at varying levels, but usually very high BP (stage 3), retinopathy with papilledema, and rapidly progressive TOD (kidneys and heart), with a fatal outcome in the absence of therapeutic intervention ( Figure 1 ). Severe BP elevation in the presence of retinal hemorrhages and exudates but no papilledema on fundoscopy is known as accelerated hypertension ( Figure 2 ). After demonstration that the clinical findings and prognosis of these two forms of hypertension are similar,^34^ the terms “malignant” and “accelerated” became interchangeable, and the World Health Organization currently uses the term accelerated-malignant to define this complication. Characteristically, malignant hypertension presents with systemic vascular changes affecting particularly the kidneys (known as malignant nephrosclerosis) and involving basically two processes: (a) proliferative endarteritis affecting small and large arterioles with intimal thickening, fragmentation, and reduplication of the internal elastic lamina and smooth muscle proliferation; the progression of this lesion, which resembles an “onion skin,” may lead to occlusion of the vessel lumen with consequent reduction in renal blood flow; (b) necrotizing changes in arterioles, especially in the glomerular hilum, and vessel wall reconstruction with eosinophilic granular material that exhibits the characteristics of fibrin (fibrinoid necrosis), causing destruction of the normal morphology and deep lumen narrowing. These changes may occur in organs other than the kidneys and are primarily responsible for the fatal complications of the disease ( Figure 3 ).^35^ The prognosis of malignant hypertension is almost always fatal if not early recognized or properly treated; in the past, the associated mortality reached 80% within 2 years.^36^ However, since the introduction of antihypertensive treatment, studies have shown that the survival of individuals with malignant hypertension has improved substantially.^37 - 39^ In a publication including almost 500 patients in Birmingham (United Kingdom), the authors reported a significant improvement in 5-year survival from 32% before 1977 to 91% in patients diagnosed between 1997 and 2006.^38^ Management of patients with malignant hypertension usually includes the use of four classes of drugs, and hypertensive complications may stabilize and, in some cases, even be reversed.

**Figure 1:** – *Normal fundoscopy (A). Fundoscopy of an individual with malignant hypertension and papilledema (B).*

**Figure 2:** – *Fundoscopy showing normal papillae, diffuse arteriolar narrowing, areas with superficial hemorrhage, and microaneurysms (grade III hypertensive retinopathy according to the Keith-Wagener classification).*

**Figure 3:** – *Anatomopathological lesions typical of accelerated-malignant hypertension. Fibrinoid necrosis of an afferent arteriole (arrow) (A). Obliterating endarteritis ("onion skin" lesions) (B).*

## 7. Stroke and Hypertensive Emergency

Stroke may present as a HE. Individuals with chronic hypertension present a right shift in the autoregulation curve for CBF causing them to tolerate substantially higher BP values without developing encephalopathy. Patients with chronic hypertension who have their BP values aggressively and rapidly reduced may present symptoms of cerebral hypoperfusion, even when the values are within the autoregulation range, as observed in normotensive individuals. Finally, patients with severe hypertension may lose the ability of autoregulation, thus presenting an increased risk of cerebral ischemia with abrupt BP reductions.^16 - 18^

### 7.1. Ischemic Stroke

In ischemic stroke, careful BP reductions of 10 to 15% are recommended at the end of the first hour after initiation of therapy and only if SBP is > 220 mmHg or DBP is > 120 mmHg.^40^ If SBP is > 180 to 230 mmHg or DBP is > 105 to 120 mmHg and the patient is not undergoing thrombolysis, the following therapy is recommended: intravenous labetalol 10 mg followed by continuous infusion at a dose of 2 to 8 mg/min; or nicardipine at the cited doses until the desired effect is obtained. If uncontrolled BP or DBP > 140 mmHg persists, intravenous sodium nitroprusside should be considered.^40^

In the case of individuals with elevated BP and indication for thrombolytic therapy with alteplase, BP should be carefully reduced until SBP < 185 mmHg and DBP < 110 mmHg before administration of the thrombolytic. If BP remains above 185/110 mmHg, thrombolytic therapy should not be administered.^40^ Labetalol is the first medication of choice, and nicardipine is the alternative therapy. A dose of intravenous labetalol of 10 to 20 mg is recommended for 1 to 2 minutes (may be repeated once). Nicardipine is recommended at the dose of 5 mg/h and administered intravenously, with dose titration of 2.5 mg/h every 5 to 15 minutes (maximum dose of 15 mg/h). During or after thrombolysis or other reperfusion therapy, BP should be maintained at or below 180/105 mmHg.^40^

### 7.2. Hemorrhagic Stroke

Treatment goals in hemorrhagic stroke are controversial.^41 - 43^ Elevation in BP is common during acute intracerebral hemorrhage and is associated with a higher risk of expansion of the hematoma, increased risk of death, and worse recovery prognosis. In this case, immediate (within 6 hours) decrease in BP to values < 140/90 mmHg has shown no benefit in the primary outcome of disability or death at 3 months, despite reducing the expansion of the hematoma and improving functional recovery.^41^ In contrast, another study has shown that a more intensive reduction in SBP is not beneficial and is associated with a greater number of adverse renal events.^42^ Thus, in individuals with hemorrhagic stroke, European guidelines recommend against immediate BP reduction for patients with SBP < 220 mmHg.^44^ In individuals with SBP ≥ 220 mmHg, careful BP reduction with intravenous therapy to achieve SBP < 180 mmHg should be considered^44^ . Labetalol, at the aforementioned doses, is the first therapeutic choice, and sodium nitroprusside and nicardipine are the alternative therapies.^1 - 4 , 28^

## 8. Acute Coronary Syndromes and Hypertensive Emergency

Epidemiological data indicate that acute coronary syndrome (ACS) is the leading cause of death and hospitalization in patients with HE. Additionally, almost 50% of all patients with hypertension admitted to the emergency room die of acute myocardial infarction (AMI) during long-term follow-up. Notably, no differences have been found when other risk factors are present, such as smoking or diabetes mellitus.^11 , 45^ Obviously, hypertension is associated with acute coronary events as a risk, atherogenic, and hemodynamic factor, imposing profound effects on cardiovascular morbidity and mortality. During a HE, increased BP causes mechanical stress and endothelial injury, leading to increased vascular permeability, activation of the coagulation cascade and platelets, fibrin deposition, and thrombosis. This process results in ischemia and release of vasoactive mediators, leading to a vicious cycle of permanent injury. The activation of the renin-angiotensin system leads to increased vasoconstriction and production of proinflammatory cytokines (tumor necrosis factor [TNF]-alpha, interleukin [IL]-6, etc.). It also increases NADPH oxidase activity and production of reactive oxygen species, causing oxidative stress. These mechanisms promote hypoperfusion, myocardial ischemia, and endothelial dysfunction, which manifest during the HE.^14 , 15^

Assessment of cardiovascular risk and investigation of comorbidities are essential in the approach to patients presenting with HE and ACS. Electrocardiography is the gold standard for the detection of ischemia or acute coronary events. Also, vital signs (BP, oxygen saturation, and heart rate) should be carefully measured during physical examination in patients with HE. Laboratory analysis includes the quantification of cardiac enzymes and determination of troponin I. In a retrospective study, patients with HC and increased cardiac troponin I (cTn-I) concentration were 2.7 times more likely to present adverse cardiovascular events and stroke at 2 years of follow-up compared with those with normal cTn-I values.^46^

Treatment of HE associated with ACS should initiate with nitroglycerin infusion. Nitroglycerin is a venodilator that reduces preload and cardiac oxygen demand. This agent is used mainly in ACS and acute edema along with other antihypertensive regimens.^47 - 49^ An alternative to nitroglycerin intolerance is the administration of dihydropyridine calcium-channel blockers (amlodipine, nicardipine), as they are useful for patients with ACS because of their beneficial effect on coronary blood flow. Alternatively, clevidipine – a short-acting calcium-channel blocker – may be administered intravenously, and since its dosing regimen is not based on weight, it allows for prolonged infusion and successful transition to oral therapy.^50^ If available, especially in ST-segment elevation ACS, primary angioplasty is the best choice for reperfusion therapy in patients with HE, as thrombolysis may increase the risk of cerebral bleeding.^47 - 49 , 51^

Beta-blockers like labetalol (a nonselective alpha-1-adrenergic receptor blocker), which reduces systemic vascular resistance while maintaining cerebral, renal, and coronary blood flow, or esmolol (a short-acting cardioselective beta-1 blocker with fast onset of action) are indicated to attenuate the increase in heart rate, reduce myocardial oxygen consumption without compromising the left ventricular diastolic filling, and improve prognosis.^28^ Additionally, BP reduction decreases the risk of pulmonary edema and the size of the infarct zone.^52^ Tolerance to higher maintenance doses of esmolol is a good predictor of results with oral beta-blocker therapy.^53^

The optimal BP value after ACS remains controversial. Several studies have shown an inverse relationship between DBP and ischemic adverse cardiac events ( *i.e.* , the lower the DBP, the higher the risk of coronary heart disease and adverse outcomes). This effect is defined as the J-curve phenomenon, which describes the shape of the relationship between BP and the risk of cardiovascular morbidity and mortality.^54^ This profile seems to be more pronounced in patients with underlying coronary artery disease.^55^

## 9. Acute Left Ventricular Dysfunction in Hypertensive Emergency

Acute left ventricular dysfunction is best known as APE. HE, acute mitral regurgitation (papillary muscle dysfunction secondary to ischemic disease or spontaneous rupture), and ACS are the most common causal factors of cardiogenic APE.^56 , 57^ About 1/3 of the patients admitted with APE and HE have preserved left ventricular function. Patients with HE presenting manifestations of APE should be managed in an intensive care unit, receive parenteral medications and monitoring, and undergo gradual BP decrease.^58^ Nitroglycerin and sodium nitroprusside are used to reduce preload and afterload. Administration of loop diuretics also decreases volume overload and helps reduce BP. The use of noninvasive continuous positive airway pressure may help reduce pulmonary edema and venous return.^28 , 59^

## 10. Acute Aortic Syndromes

Acute aortic syndrome (AAS), a term currently comprising aortic dissection (AD), intramural hematoma (IMH), and penetrating atherosclerotic ulcerations (PAU), has an incidence that ranges from 3.5 to 6.0 per 100,000 patients/year.^60^ Given its high mortality rate, AAS should be considered and promptly diagnosed in patients with acute chest or back pain, especially if associated with hypertension. Computed tomography, magnetic resonance imaging, and transesophageal echocardiography are reliable imaging tests to diagnose AAS, while measurement of serum D-dimer has shown 51.7 to 100% sensitivity and 32.8 to 89.2% specificity in six studies.^61^

Of all AAS types, AD is the most common (85 to 95%), followed by IMH (0 to 25%) and PAU (2 to 7%).^61^ According to the Stanford classification, AAS is divided into type A, which involves the ascending aorta, and type B, which does not involve this segment. In contrast, the DeBakey classification divides AAS into type I, which involves at least the ascending aorta and the aortic arch and often also the descending aorta; type II, which is confined to the ascending aorta; and type III, which originates in the distal descending aorta and affects the left subclavian artery.^60^ AAS may be associated with several risk factors including the male sex, advanced age, first-degree relatives with a history of AAS, hypertension, dyslipidemia, smoking, illicit drug use, history of major vascular arteritis ( *e.g.* , Takayasu arteritis), collagen vascular disease (like Marfan’s, Loeys-Dietz, and Ehlers-Danlos syndrome), blunt trauma from motor vehicle accident or vertical fall, arterial instrumentation for diagnostic or therapeutic purposes, or hereditary mutations in genes encoding proteins involved with vascular integrity (such as mutation in the *ACTA2* gene).^60^

### 10.1. Treatment

Treatment of AAS requires a multidisciplinary approach involving clinical, endovascular, and surgical interventions.^62^ Type A ADs have a poor prognosis and an overall in-hospital mortality of 30%, with a mortality increase of 1 to 2% per hour of progression.^63^ Without intervention, the mortality is about 58%, compared with 26% with surgical intervention.^63^ Open surgery is the ideal treatment for type A AAS (ascending aorta), and thoracic endovascular aortic repair is best suited to treat type B AAS (descending aorta).^64 - 66^ Endovascular surgery has been shown to be better than medical treatment (97% *vs.* 43%) considering the favorable aortic remodeling, false lumen thrombosis, and absence of aortic dilation or rupture.^66^

Initial management of AD involves pain control and use of antihypertensive agents. Intravenous beta-blockers (metoprolol, esmolol, or labetalol) should be administered to reduce wall stress, lowering heart rate and BP and maintaining adequate cerebral, coronary, and renal perfusion.^60^ Administration of beta-blockers should be completed before BP reduction with afterload reducing agents. Guidelines recommend a SBP reduction to 100 to 120 mmHg and a heart rate below 60 bpm.^65^ In case of intolerance to beta-blockers, non-dihydropyridine calcium-channel blockers (verapamil or diltiazem) should be used.^67^ After proper beta blockade, afterload should be reduced. Although angiotensin-converting enzyme inhibitors (ACEIs) have not shown significant benefits in terms of mortality, they have been used as adjuvant agents to reduce BP.^68^ Sodium nitroprusside may also be used after beta blockade since, as monotherapy, this agent may increase shear stress of the aortic wall resulting in progression of the dissection.^60^ To date, there is no known indication for early platelet blockade in AD control.^60^ Several studies have shown that the use of statins reduces the growth rate of abdominal aortic aneurysm (AAA) and decreases the likelihood of recurrent rupture after repair.^69^ Still, the role of statins in AAS is unclear.^69^ Effective pain management with morphine sulfate, fentanyl, or opiate should be implemented.^60^

## 11. Hypertensive Emergencies During Pregnancy

Hypertension is the most common medical problem in pregnancy, manifesting in up to 10% of all pregnancies and accounting for about 25% of prenatal hospital admissions; it is also an important cause of maternal and fetal morbidity and mortality. Women with hypertension during pregnancy are at higher risk for future hypertensive disease, stroke, and coronary artery disease.^70 , 71^ The definition of hypertension in pregnancy follows the same criteria of the Brazilian Guideline of Arterial Hypertension, i.e., BP ≥ 140/90 mmHg. Hypertension during pregnancy is considered severe when SBP values are ≥ 160 to 170 mmHg and DBP are ≥ 110 mmHg.^72^ Thus, hypertension may precede (in this case, chronic hypertension) or develop during the course of pregnancy (preeclampsia/eclampsia/gestational hypertension), characterizing four different categories of hypertension:^70 - 72^

1. Chronic hypertension begins before pregnancy or is diagnosed before the 20th week of gestation. Only 20 to 25% of the cases of chronic hypertension in pregnancy progress to preeclampsia.
2. Gestational hypertension is the most common disorder (10% of the cases occur in primiparous women; 20 to 25% of the cases overlap chronic hypertension). It develops after the 20^th^gestational week and is not accompanied by proteinuria. BP returns to normal values 1 to 2 weeks after delivery. Progresses with a favorable maternal and fetal prognosis.
3. Preeclampsia/eclampsia. Preeclampsia (PE), a process specific of pregnancy, is defined by hypertension that appears after the 20^th^gestational week and presents with proteinuria (> 300 mg/24 hours or protein/creatinine ratio > 300 mg/g), edema, and sometimes abnormal coagulation and liver function. Preeclampsia can progress rapidly to eclampsia, a clinical condition characterized by tonic-clonic seizures preceded by severe hypertension, headache, and hyperreflexia. Cerebral hemorrhage is the most serious complication, with a high rate of maternal mortality. Proteinuria and elevated BP should return to normal within 12 weeks after delivery.
4. Chronic hypertension with preeclampsia/overlapping eclampsia. This condition should be suspected in the presence of microalbuminuria (30 to 300 mg in 24-hour urine or 30 to 300 mg/g albumin/creatinine ratio in spot urine), increase in preexisting proteinuria, clinical or laboratory abnormality characteristic of preeclampsia, or elevation in preexisting BP levels after the 20^th^gestational week in a patient with chronic hypertension.

### 11.1. Treatment

The two main key points in the treatment of HC in pregnancy are (1) stabilization of the mother, including the use of antihypertensive medications that are safe and appropriate for use in pregnancy, and delivery recommendation; and (2) fetal well-being, which must be confirmed by fetal monitoring and ultrasound.

Pharmacological treatment should be initiated at BP levels > 150/100 mmHg, aiming at maintaining the levels at 130 to 150/80 to 100 mmHg (degree of recommendation [DR]: IIa; level of evidence [LE]: B). In patients with preeclampsia in stable clinical condition without the need for immediate delivery, oral antihypertensive treatment is indicated.^72^ In Brazil, the oral medications that are usually administered are methyldopa, hydralazine, calcium-channel antagonists (long-acting nifedipine, amlodipine), and beta-blockers (preferably pindolol). Pregnant women with chronic hypertension may continue the use of thiazides, as long as they do not promote volume depletion.^73^ The use of renin-angiotensin system blockers is contraindicated in pregnancy (DR: I; LE: B).^72^

Urgent pharmacological treatment is indicated in severe hypertension (SBP > 155 to 160 mmHg) and in the presence of premonitory signs (DR: I; LE: B). Intravenous hydralazine is recommended (5 mg, repeat 5 to 10 mg every 30 minutes to a maximum of 20 mg). Sodium nitroprusside may be considered for urgent BP control, especially in the presence of APE and severe and refractory hypertension.^72^

Magnesium sulfate is the medication of choice for both treatment and prevention of seizures during eclampsia. The patient should be monitored in terms of urine output, patellar reflexes, respiratory rate, and oxygen saturation. Plasma magnesium should be maintained between 4 and 7 mEq/L and measured in the occurrence of renal disease. If magnesium sulfate intoxication is suspected, calcium gluconate should be administered.^70 , 71^

## 12. Adrenergic Emergencies

Neuroendocrine tumors associated with sympathetic tissue with the potential to secrete catecholamines are rare and include pheochromocytomas (adrenal medulla) and paragangliomas (non-adrenal tissue). Diagnosis, location, and anatomical delineation of these tumors involve measurement of catecholamines and their metabolites in blood and urine, computed tomography and/or magnetic resonance imaging, and metaiodobenzylguanidine (I^123^) scintigraphy. Symptoms may occur at any stage of life, are nonspecific, and depend on the release of catecholamines into the bloodstream; BP elevation, palpitations, and headache may occur. Surgical removal of these tumors is always indicated to cure or prevent cardiovascular disease secondary to catecholamine excess.^74^ BP in these patients may be sustained or paroxysmal, and a marked increase in BP may characterize an impending life-threatening HE. This occurs by activation of alpha receptors by catecholamines. The Brazilian Guideline on Hypertension recommends a diagnostic flowchart for neuroendocrine tumors (pheochromocytoma and paragangliomas), which is shown in Table 4 .^75^Figure 4 shows the imaging methods used for diagnostic confirmation in the occurrence of an abnormal biochemical test.

**Table 4:** – Flowchart from the 7th Brazilian Guideline of Arterial Hypertension for clinical and laboratory diagnosis of cases of pheochromocytoma and paraganglioma

Clinical findings	Suspected diagnosis	Additional studies
- Paroxysmal hypertension with headache, sweating, and palpitations	Pheochromocytoma	- Free plasma metanephrines
- Resistant hypertension		- Urinary metanephrines and serum catecholamines
		- Imaging tests

**Figure 4:** – *Imaging methods for diagnostic confirmation of pheochromocytoma.*

Whole-body scintigraphy is obtained to identify the location of extra-adrenal neuroendocrine tumors (paragangliomas). This test is recommended in cases of abnormal biochemical tests and negative imaging tests. It should always be performed after verification and discontinuation of medications that may interfere with their interpretation (sympathomimetics, calcium-channel blockers, cocaine, antidepressants, and labetalol), which should be suspended 14 days prior to the test. Whole-body scintigraphy is contraindicated during pregnancy.^76^ After a diagnosis of neuroendocrine tumor, the proposed treatment is always surgical, preceded by pharmacological preparation and hydration to prevent or mitigate the occurrence of HC or hypotension during surgery ( Table 5 ).^76^ In this situation, intravenous antihypertensive medications are administered (initially alpha-blockers and later beta-blockers). Continuous infusion of sodium nitroprusside (0.25 to 10 mg/kg/min) or phentolamine (continuous infusion of 1 to 5 mg with a maximum dose of 15 mg) may be used with markedly increased BP.^75 - 77^

**Table 5:** – Preoperative care in cases of pheochromocytoma

High-sodium diet and hydration (lacks evidence):
- Saline infusion during surgery (1 to 2 L)
- Revert volume contraction
- Prevent hypotension

**Pharmacological preparation:**

- Alpha-adrenergic blockade
- Beta-blockers
- Calcium-channel blockers
- No evidence regarding target blood pressure

**Laparoscopic adrenalectomy (most cases):**

- For paragangliomas (minority)

**Open adrenalectomy (for paragangliomas):**

- For pheochromocytoma (minority)

## 13. Illicit Drugs and Hypertensive Emergency

In the emergency room, patients with HC and sympathetic hyperactivity should raise suspicion of amphetamine or cocaine intoxication, as well as abusive use of other drugs like serotonin reuptake inhibitors, monoamine oxidase inhibitors, and use of cytotoxic or antiangiogenic medications.^52^

Cocaine has multiple cardiovascular and hematological effects that contribute to BP elevation, development of myocardial ischemia, and/or AMI due to coronary vasoconstriction. Cocaine, even in small doses, blocks norepinephrine and dopamine reuptake in presynaptic adrenergic terminals, causing catecholamine accumulation in the postsynaptic receptor, thus acting as a powerful sympathomimetic agent.^78^ As a result, cocaine causes a dose-dependent increase in heart rate and BP.^79^ In addition, cocaine use may reduce left ventricular function associated with increased parietal stress at the end of systole and increased oxygen demand. The chronotropic effects of cocaine use are intensified by alcohol consumption.^80^ Cocaine-induced vasoconstriction is secondary to stimulation of alpha-adrenergic receptors in the smooth muscle cells of the coronary circulation. This drug also increases the release of endothelin-1^81^ and decreases the bioavailability of nitric oxide, promoting BP elevation.^82^ Treatment with benzodiazepines is initially indicated. When BP reduction is required, a competitive intravenous alpha-blocker agent is indicated (phentolamine). Alternatively, nicardipine or sodium nitroprusside may be considered.^83^ Clonidine may also be considered because of its sedative effect in addition to sympatholytic action.

In ACS, treatment with nitroglycerin and aspirin is recommended concomitantly with benzodiazepines. In the presence of ACS with tachyarrhythmias, non-dihydropyridine calcium-channel blockers (diltiazem and verapamil) are recommended. Beta-blockers (including labetalol) are contraindicated since these agents are unable to reduce the coronary vasoconstriction.^84^ Nicardipine may also be a good alternative for patients with HE induced by cytotoxic or antiangiogenic drugs.

## 14. Postoperative Hypertensive Emergency Following Vascular Surgery

The concept of “postoperative hypertensive emergency” differs from that of ambulatory hypertensive emergency/urgency because of the occurrence of this unique clinical situation in an atypical (postoperative) setting. Notably, moderately elevated BP values in the postoperative setting may require immediate treatment.^85^

Postoperative hypertensive emergency (POHE) is arbitrarily defined as elevation of SBP to levels > 190 mmHg and/or DBP to levels > 100 mmHg confirmed in two consecutive readings during the immediate postoperative period.^86^ A 40 to 50 mmHg elevation in SBP or increase in BP values greater than 20% in relation to baseline values may also characterize postoperative hypertension.^87^ This increase in BP values usually begins 10 to 20 minutes after surgery and can last up to 4 hours. The pathophysiology of POHE in patients previously normotensive is associated with peripheral vasoconstriction, catecholamine release, reduced baroreceptor sensitivity, central adrenergic activation, vasopressin release, stimulation of the renin-angiotensin system with consequent angiotensin II production, release of inflammatory cytokines (IL-6), and sodium retention. All these changes result in vasoconstriction, increase in afterload and SBP/DBP, and tachycardia. If left untreated, postoperative hypertension increases the risk of myocardial ischemia, AMI, APE, stroke, and bleeding, as well as postoperative mortality.^88 , 89^

POHE occurs in 40 to 80% of the patients undergoing carotid endarterectomy or open cardiac surgery, 57% of the patients undergoing abdominal aortic surgery and 29% of those undergoing peripheral vascular surgery.^90 - 92^ In particular, acute and severe hypertension with SBP elevation > 220 mmHg may occur in 9% of the individuals undergoing carotid endarterectomy.^93^ This manifestation, which may be transient, is related to carotid sinus manipulation and may cause hematoma, myocardial ischemia, and cerebral hyperperfusion with consequent neurological damage.^94^ Other proposed mechanisms include iatrogenic denervation,^95^ decreased baroreflex activity,^96^ reduced carotid sinus sensitivity, and increased production of cerebral renin and/or catecholamines.^97 , 98^

HE may also occur after surgical correction of aortic coarctation. The etiology is multifactorial and includes changes in the baroreceptor reflex, activation of the sympathetic system and renin-angiotensin system, and expansion of the extracellular volume.^99^ The stimulation of sympathetic nerve fibers located in the middle layer and adventitia of the aortic isthmus has two effects, both resulting in hypertension. Initially, peripheral release of norepinephrine occurs, with consequent vasoconstriction and BP elevation. Next, stimulation of juxtaglomerular cells occurs, releasing renin and promoting additional hypertension. Secondarily, increased renin production causes blood shunting from the mesenteric arteries, thus triggering abdominal symptoms in the so-called post-coarctectomy syndrome.^100^

Before initiating antihypertensive pharmacological treatment, reversible causes of postoperative hypertension should be investigated, such as pain, hypoxia, hypercapnia, agitation, bladder distension, and hypervolemia.^101^ Proper analgesia and sedation are considered to be requirements before the initiation of antihypertensive therapy.^102^ When POHE is present, the distinction between emergency and urgency is mandatory.^1 - 4^ The therapeutic goal is not necessarily to normalize BP but to interrupt the vascular injury and reverse the pathological process. Progressive BP reductions, as reported in the general principles of HE treatment, should be achieved.^1^
